# De-ubiquitination of SAMHD1 by USP7 promotes DNA damage repair to overcome oncogenic stress and affect chemotherapy sensitivity

**DOI:** 10.1038/s41388-023-02667-w

**Published:** 2023-04-20

**Authors:** Jingwei Liu, Tingting Zhou, Xiang Dong, Qiqiang Guo, Lixia Zheng, Xiaoxun Wang, Naijin Zhang, Danni Li, Ling Ren, Fei Yi, Ying Zhang, Ziwei Li, Xiwen Wang, Chengsi Deng, Chunlu Li, Hongde Xu, Yi Guan, Xiaoman Li, Yang Yu, Wendong Guo, Zhuo Wang, Bo Jiang, Xuan Wu, Ning Bai, Yanling Feng, Mengtao Ma, Qingquan Kong, Jiayi Wei, Zhenshuang Wang, Hao Li, Songming Lu, Liangzi Cao, Yutong Xiao, Xiaoyu Song, Zhenning Wang, Chengzhong Xing, Liu Cao

**Affiliations:** 1 The College of Basic Medical Science, China Medical University, Shenyang, Liaoning Province China; 2 Key Laboratory of Cell Biology of Ministry of Public Health, Key Laboratory of Medical Cell Biology of Ministry of Education, Key Laboratory of Precision Diagnosis and Treatment of Gastrointestinal Tumors (China Medical University), Ministry of Education, Liaoning Province Collaborative Innovation Center of Aging Related Disease Diagnosis and Treatment and Prevention, China Medical University, Shenyang, Liaoning Province China; 3 Department of Developmental Cell Biology, Key Laboratory of Cell Biology, Ministry of Public Health, and Key Laboratory of Medical Cell Biology, Ministry of Education, China Medical University, Shenyang, Liaoning Province China; 4 Department of Anus and Intestine Surgery, First Affiliated Hospital of China Medical University, Shenyang, Liaoning Province China; 5 Department of Laboratory Medicine, The First Affiliated Hospital of China Medical University, Shenyang, Liaoning Province China

**Keywords:** Ubiquitylation, Cancer therapeutic resistance

## Abstract

Oncogenic stress induces DNA damage repair (DDR) that permits escape from mitotic catastrophe and allows early precursor lesions during the evolution of cancer. SAMHD1, a dNTPase protecting cells from viral infections, has been recently found to participate in DNA damage repair process. However, its role in tumorigenesis remains largely unknown. Here, we show that SAMHD1 is up-regulated in early-stage human carcinoma tissues and cell lines under oxidative stress or genotoxic insults. We further demonstrate that de-ubiquitinating enzyme USP7 interacts with SAMHD1 and de-ubiquitinates it at lysine 421, thus stabilizing SAMHD1 protein expression for further interaction with CtIP for DDR, which promotes tumor cell survival under genotoxic stress. Furthermore, SAMHD1 levels positively correlates with USP7 in various human carcinomas, and is associated with an unfavorable survival outcome in patients who underwent chemotherapy. Moreover, USP7 inhibitor sensitizes tumor cells to chemotherapeutic agents by decreasing SAMHD1 in vitro and in vivo. These findings suggest that de-ubiquitination of SAMHD1 by USP7 promotes DDR to overcome oncogenic stress and affect chemotherapy sensitivity.

## Introduction

Balance between DNA damage and DNA damage repair (DDR) enable cells to maintain genome integrity against endogenous and exogenous insults [1]. An underlying hallmark of cancers is their genomic instability, which is characterized by a greater propensity to accumulate DNA damage [2]. As a result, cancer cells demonstrate increased genomic instability and a greater dependency on DDR pathways to overcome frequent DNA damage [3]. A variety of chemotherapeutics induce DNA damage to kill tumor cells. However, activated DDR in cancer cells can repair DNA damage induced by genotoxic insults thus leading to chemotherapy insensitivity [4]. Therefore, understanding the key regulators and mechanisms orchestrating DDR may potentially elucidate mechanisms of tumorigenesis and provide novel therapeutic target for cancer patients [5].

SAMHD1, an important dNTP hydrolase, maintains nucleic acid metabolism and genome stability by regulating the abundance of the dNTP pool in cells [6, 7]. The enzyme was first found to be correlated with Aicardi-Goutières syndrome, suggesting that SAMHD1 participates in immune regulation and innate immunity [8, 9]. In addition, SAMHD1 restricts DNA viruses and retroviruses by virtue of its dNTP hydrolysis ability, and this direct action on viral genetic material has a broad spectrum [10]. At present, most of the studies focused on the importance of antiviral role of SAMHD1 [11], while the potential role of SAMHD1 in tumorigenesis and therapy remains largely elusive. Recently, SAMHD1 was found to promotes DNA end resection to facilitate DNA double-strand break (DSB) repair by interacting with CtIP independent of its dNTP hydrolysis ability [12]. DSBs are the most cytotoxic type of DNA damage, as unrepaired or inappropriate repair of DSBs inevitably causes mutations or chromosomal aberrations [13]. Considering its role in DNA damage repair, SAMHD1 might be implicated in tumorigenesis and response to chemotherapy in cancer.

Emerging evidence have indicated that multiple post-translational protein modifications including ubiquitination are actively involved in the DDR process [14, 15]. Deubiquitinating enzyme (DUB) is responsible for removing the ubiquitination modification of the substrate, maintaining the stability of the substrate, and preventing it from degradation by the proteasome [16]. Ubiquitin-specific proteases (USPs) constitute the largest DUB subfamily, of which USP7 is a key deubiquitinating enzyme stabilizing multiple substrates through deubiquitination, thereby regulating a variety of cellular processes including immune response, virus replication, and cancer [17–20]. USP7 has previously been reported to regulate DDR proteins: USP7 modulates the engagement of the MRN-MDC1 complex and the consequent recruitment of the downstream factors at DNA lesions; [21] USP7 is essential for maintaining stability of Rad18 [22] and RNF168 [23] for DDR; In addition, USP7 controls the stability of Chk1, an essential checkpoint kinase in DDR [24].

In this study, we have demonstrated that USP7 interacts with SAMHD1 at the HD domain and deubiquitinates SAMHD1 at K421, thus stabilizing SAMHD1 by reducing its degradation via proteasome pathway. USP7-stabilized SAMHD1 promotes DDR by interacting with DSB repair initiator CtIP, therefore repairing DNA damage induced by reactive oxygen species (ROS) or genotoxic insults. Attesting to the function, both USP7 and SAMHD1 proteins were highly expressed in human carcinomas of various organs and their expressions were positively correlated. Most importantly, the USP7 inhibitor sensitizes tumor cells to chemotherapy by decreasing SAMHD1, which implies the potential of targeting USP7-SAMHD1-CtIP axis to improve chemosensitivity.

## Results

### USP7 interacts with SAMHD1 at the HD domain and stabilizes its protein expression

**Fig. 1:**
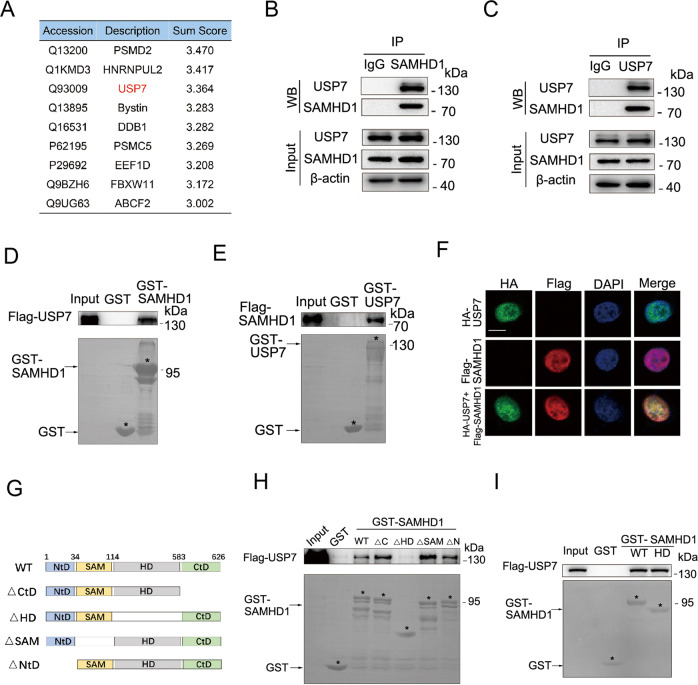
USP7 interacts with SAMHD1 at the HD domain. **A** Flag-tagged SAMHD1 was transfected into the HEK293 cells and immunoprecipitated with an anti-Flag antibody. SAMHD1 was purified with a Flag peptide followed by mass spectrometry analysis. **B** The lysates of the HCT116 cells were immunoprecipitated with the IgG control or anti-SAMHD1 antibody followed by immunoblotting with USP7 and SAMHD1 antibodies. **C** The lysates of the HCT116 cells were immunoprecipitated with the IgG control or anti-USP7 antibody followed by immunoblotting with USP7 and SAMHD1 antibodies. **D**, **E** SAMHD1 interacts with USP7 in vitro demonstrated by a GST pull-down assay. Recombinant human SAMHD1 or USP7 was incubated with bacterially expressed GST-USP7 or GST-SAMHD1. **F** SAMHD1 colocalized with USP7 in Hela cells by immunofluorescence assay. Scale bar, 10 μm. **G** Schematic plot for constructing truncated plasmids of SAMHD1 lacking different domains including NtD (1-34), SAM (35-114), HD (115-583), or CtD (584-626), respectively. **H** USP7 could not interact with HD domain-deleted SAMHD1. Truncated plasmid of SAMHD1 lacking different domains including NtD, SAM, HD, or CtD were constructed for GST pull-down assay with recombinant USP7, respectively. **I** USP7 interacts with the HD domain of SAMHD1.

**Fig. 2:**
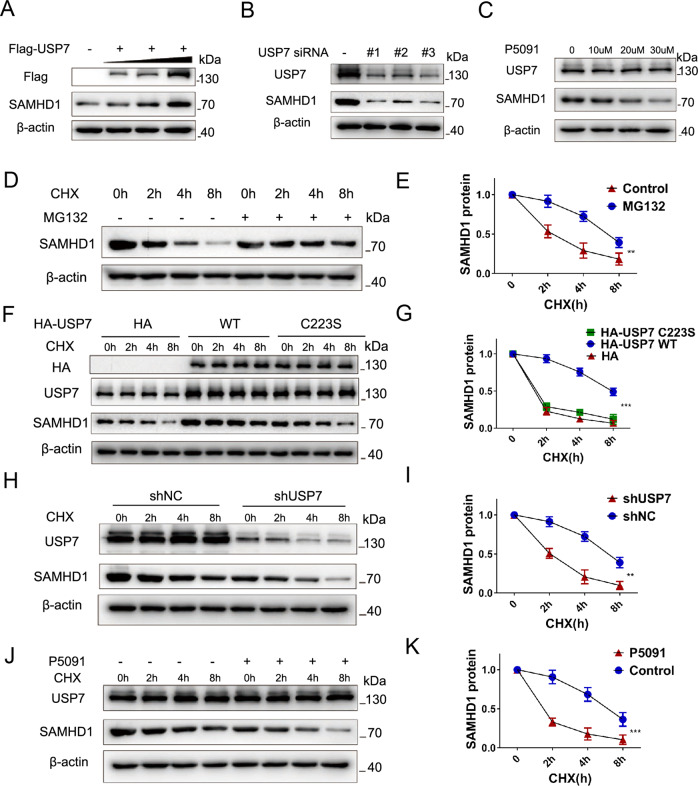
USP7 stabilizes the expression of SAMHD1. **A** Western blot analysis demonstrating gradually increased SAMHD1 protein levels with increased amounts of Flag-USP7 plasmids transfected. **B** The effect of three target sequences of siRNA-USP7 on the expression of SAMHD1 48 h after transfection. **C** Western blot analysis of SAMHD1 with or without P5091 treatment for 24 h at different concentrations. **D**, **E** Levels of SAMHD1 expression after different durations of CHX (20 μM) administration with or without MG132 (20 μM) treatment. Data are shown as mean ± SEM (**P < 0.01). **F**, **G** SAMHD1 expression after different durations of CHX (20 μM) administration in the cells 48 h after transfected with HA vector, HA-USP7 WT, or HA-USP7 C223S mutant plasmids. Data are shown as mean ± SEM (***P < 0.001). **H**, **I** SAMHD1 expression after different durations of CHX (20 μM) administration, with or without USP7 knockdown. Data are shown as mean ± SEM (**P < 0.01). **J**, **K** SAMHD1 expression after different durations of CHX (20 μM) administration, with or without P5091 (20 μM) pretreatment. Data are shown as mean ± SEM (***P < 0.001).

Although the ubiquitination modification of SAMHD1 has been widely reported [25–27], the deubiquitinating enzyme (DUB) of SAMHD1 is still unknown. We next performed mass spectroscopy to identify potential molecules interacting with SAMHD1 (Fig. 1A). The observations in this regard suggested that USP7 might be a specific DUB for SAMHD1, a notion supported by the subsequent co-immunoprecipitation assays in HCT116 (Fig. 1B, C), Hela (Fig. S1A), HEK293 (Fig. S1B), A549 (Fig. S1C, D) and TPC-1 (Fig. S1E, F) cells. The USP7-SAMDH1 interaction was confirmed by the in vitro glutathione-S-transferase (GST)-pulldown assays (Fig. 1D, E). Furthermore, co-localization of USP7 and SAMHD1 were observed by confocal microscopy in Hela cells (Fig. 1F). To identify the specific USP7 binding domain in SAMHD1, we constructed different GST-SAMHD1 fusion proteins lacking each domain for the subsequent GST-pulldown assays (Fig. 2G). To that end, GST-SAMHD1 lacking the HD domain failed to interact with USP7 (Fig. 2H) while the HD domain of SAMHD1 interact with USP7 (Fig. 2I), suggesting that it is essential for USP7-SAMHD1 interaction.

DUB deubiquitinates substrate and maintains the stability of the substrate, preventing it from degradation by the proteasome [28]. We next examined the effect of USP7 on SAMHD1 stability by overexpressing USP7 in HCT116 cells. To that end, SAMHD1 protein levels were gradually increased with the increased amount of USP7 plasmids transfected (Fig. 2A). Consistent results were found in lung cancer A549 and thyroid cancer TPC-1 cells (Fig. S1G, H). In contrast, all three different siRNAs for USP7 reduced SAMHD1 expression in HCT116 cells (Fig. 2B). P5091, a specific USP7 inhibitor, decreased SAMHD1 levels in both concentration and time dependent manners in HCT116 (Fig. 2C; Fig. S1K) and H1299 (Fig. S1J) cells, respectively. Proteasome inhibitor MG132 prolonged the half-life of SAMHD1 protein under CHX treatment, suggesting that ubiquitin–proteasome pathway is a key manner for SAMHD1 protein degradation (Fig. 2D, E). Overexpressing USP7 wildtype also lengthened the half-life of SAMHD1 protein, in contrast to the effect of USP7 C223S, an inactivation mutant of the deubiquitination enzyme (Fig. 2F, G). Conversely, stable knockdown of USP7 (Fig. 2H, I) or treatment with P5091 shortened the half-life of SAMHD1 (Fig. 2J, K).

### USP7 deubiquitinates SAMHD1 K48-linked polyubiquitination at K421

**Fig. 3:**
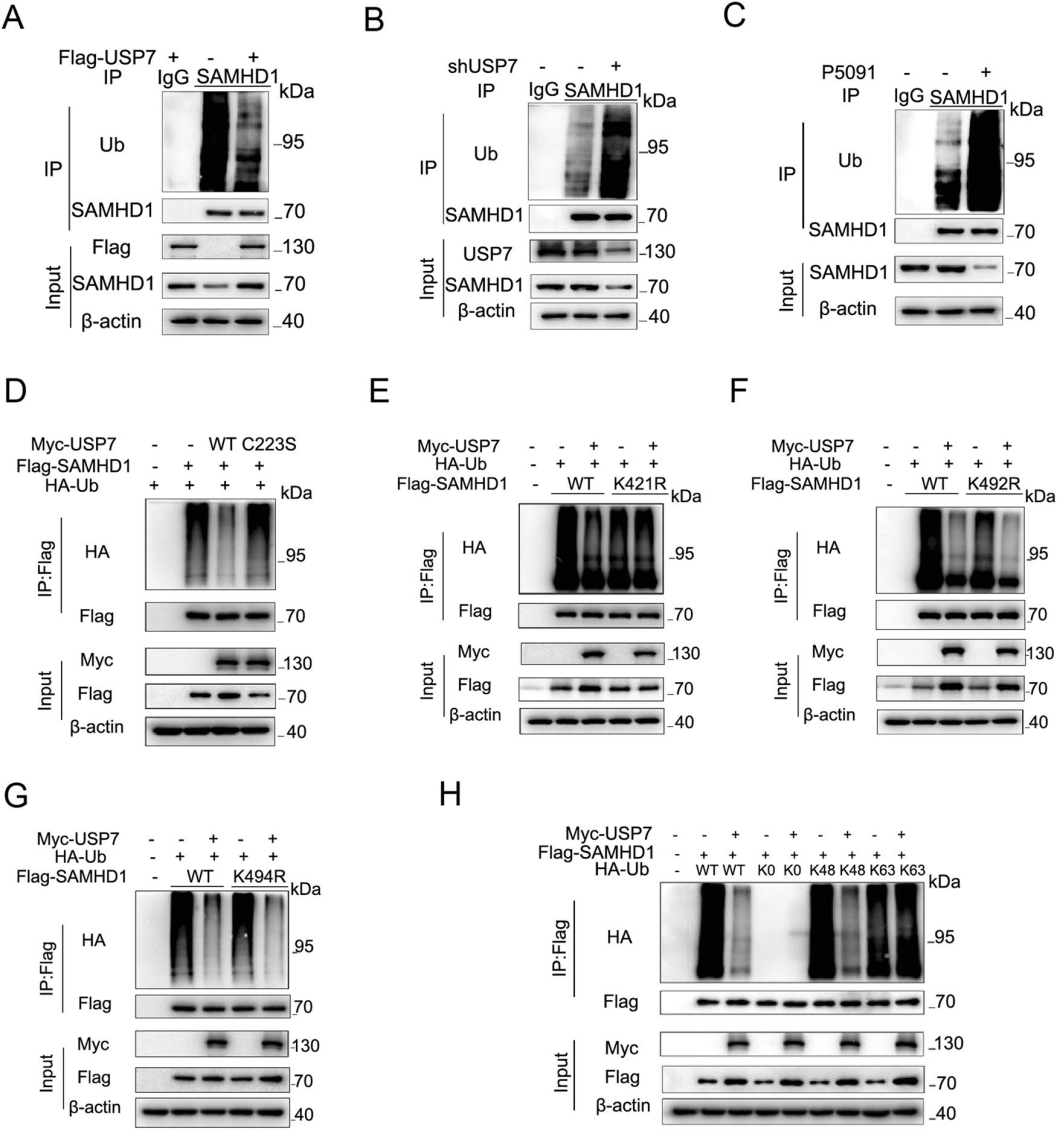
USP7 deubiquitinates SAMHD1 K48-linked polyubiquitination at K421. **A** The HCT116 cells were lysed 48 h after transfection with or without Flag-USP7, and then immunoprecipitated with the anti-SAMHD1 antibody or IgG control, followed by immunoblotting with the ubiquitin antibody. **B** The lysates of HCT116 cells with or without USP7 knockdown were immunoprecipitated with the anti-SAMHD1 antibody or IgG control, followed by immunoblotting with the ubiquitin antibody. **C** The lysates of HCT116 cells with or without P5091 (20 μM) pretreatment for 24 h were immunoprecipitated with the anti-SAMHD1 antibody or IgG control, followed by immunoblotting with the ubiquitin antibody. **D** The HCT116 cells were lysed 48 h after transfection with Flag-SAMHD1, HA-Ub, and Myc/Myc-USP7 WT/Myc-USP7 C223S, respectively, and then immunoprecipitated with the Flag antibody, followed by immunoblotting with the HA antibody. **E-G** The lysates of HCT116 cells were lysed 48 h after transfection with Flag-SAMHD1 WT/Flag-SAMHD1 K421R/Flag-SAMHD1 K492R/Flag-SAMHD1 K494R, HA-Ub, and Myc/Myc-USP7, and then immunoprecipitated with the Flag antibody, followed by immunoblotting with the HA antibody. **H** The HCT116 cells were lysed 48 h after transfection with Flag-SAMHD1, Myc-USP7, and HA-Ub WT/K0/K48/K63, respectively, and then immunoprecipitated with the Flag antibody, followed by immunoblotting with the HA antibody.

To explore the effect of USP7 on SAMHD1 ubiquitination, we next overexpressed Flag-USP7. As a result, deubiquitination of SAMHD1 protein was found in HCT116 (Fig. 3A), HEK293 (Fig. S1L), A549 (Fig. S1M), and TPC-1 (Fig. S1N) cells, respectively. In contrast, stable knockdown of USP7 induced ubiquitination of SAMHD1 protein in HCT116 cells (Fig. 3B). Administration of P5091 significantly increased ubiquitination of SAMHD1 protein in HCT116 (Fig. 3C), SW480 (Fig. S1O), Hela (Fig. S1P) and HEK293 (Fig. S1Q) cells, respectively. In addition, overexpressing USP7 deubiquitinating enzyme inactivation mutant plasmid C223S failed to deubiquitinate SAMHD1 (Fig. 3D).

We next sought to identify the specific deubiquitination sites of SAMHD1 protein by USP7 via ubiquitination site mass spectrometry, which is a approach to indicate ubiquitination sites by high throughput screening for each potential lysine. The study to that end revealed K421, K492, K494 as potential ubiquitination sites (Fig. S2A-C). Co-immunoprecipitation assays showed decreased ubiquitination when lysine (K) K421 or K492 of SAMHD1 was substituted by arginine (R) (Fig. S2E). Further experiments demonstrated that SAMHD1 K492R and K494R, but not K421R, could be deubiquitinated by USP7, indicating that K421 is the specific site for USP7 deubiquitinating SAMHD1 (Fig. 3E-G). K421 is evolutionarily conservative across different species (Fig. S2D). In addition, USP7 overexpression does not significantly affect SAMHD1 K421R stability (Fig. S2F); while USP7 siRNA does not significantly affect SAMHD1 K421R stability (Fig. S2G). SAMHD1 has previously been shown to be ubiquitinated at K622 by TRIM21 and also ubiquitinated by CRL4/DCAF1, both of which target SAMHD1 for proteasomal degradation. We suggested that K421 is not the specific site for these E3 ligases, and USP7 could not deubiquitinate K622 site (Fig. S2H-J). Lastly, we transfected different HA-Ub plasmids including WT, K0, K48 and K63 along with USP7 and SAMHD1. To that end, USP7 specifically removes K48-linked polyubiquitination of SAMHD1 (Fig. 3H).

### SAMHD1 promotes cell survival and reduces apoptosis under genotoxic insults

**Fig. 4:**
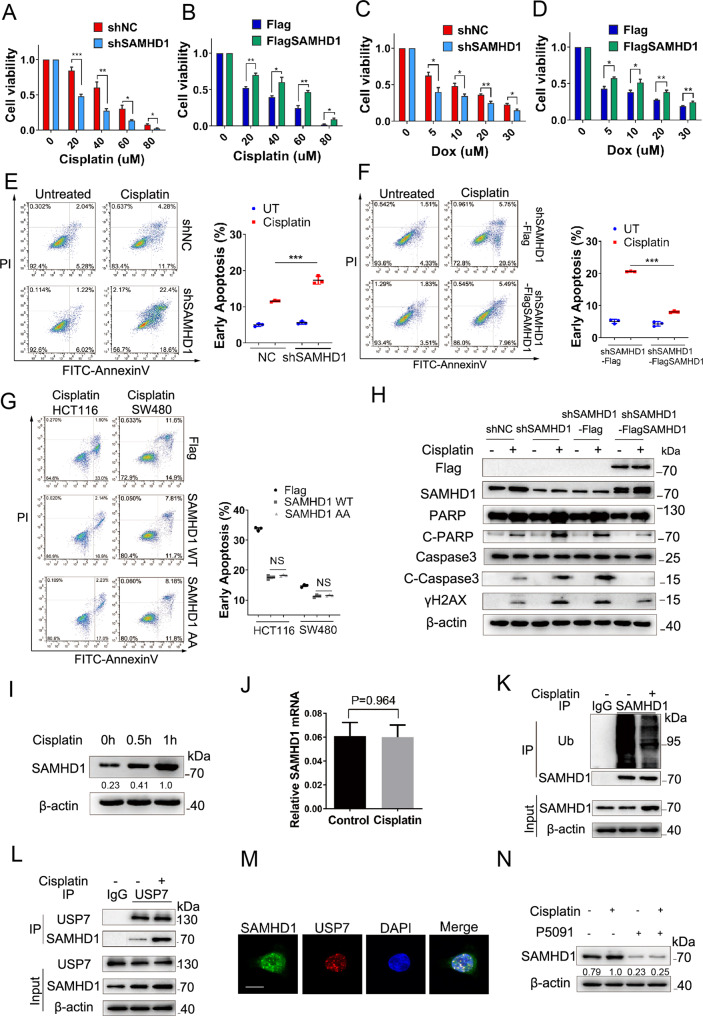
SAMHD1 increases cell survival and reduces apoptosis under genotoxic insults. **A-D** HCT116 shNC and shSAMHD1 cells or shSAMHD1-Flag and shSAMHD1-Flag-SAMHD1 cells were treated with cisplatin or doxorubicin at different concentrations for 24 h. Cell viability was assessed by CCK8 assay. **E**, **F** HCT116 shNC and shSAMHD1 cells or shSAMHD1-Flag and shSAMHD1-Flag-SAMHD1 cells were treated with cisplatin (20 μM) for 24 h followed by staining with PI and FITC-Annexin V, and analyzed by fluorescence-activated cell sorting (FACS). Scatter graph represents percentage of apoptotic cells from three independent experiments. ***P < 0.001. **G** HCT116 and SW480 shSAMHD1-Flag, shSAMHD1-Flag-SAMHD1 WT, and shSAMHD1-Flag-SAMHD1 H206A/D207A cells were treated with cisplatin (20 μM) for 24 h followed by staining with PI and FITC-Annexin V, and analyzed by FACS. Scatter graph represents percentage of apoptotic cells from three independent experiments. NS, no significance. **H** HCT116 shNC and shSAMHD1 cells or shSAMHD1-Flag and shSAMHD1-Flag-SAMHD1 cells were treated with cisplatin (5 μM) for 12 h followed by western blot analysis of Flag, SAMHD1, PARP, Cleaved-PARP, Caspase 3, Cleaved-Caspase 3, and γH2AX. **I** Western blot analysis of SAMHD1 in HCT116 cells with or without cisplatin (5 μM) treatment. **J** Quantitative PCR for SAMHD1 mRNA in HCT116 cells with or without cisplatin (5 μM) treatment for 1 h. **K** The lysates of HCT116 cells in with or without 1 h of cisplatin (5 μM) stimulation were immunoprecipitated with the IgG control or anti-SAMHD1 antibody, followed by immunoblotting with the ubiquitin antibody. **L** The lysates of HCT116 cells with or without cisplatin (5 μM) stimulation for 1 h were immunoprecipitated with the anti-USP7 antibody followed by immunoblotting with the SAMHD1 and USP7 antibodies. **M** HCT116 cells were stimulated with cisplatin (5 μM) for 4 h, and then stained with the anti-SAMHD1 and anti-USP7 antibodies, DAPI, respectively, for immunofluorescence analysis. Scale bar, 10 μm. **N** Western blot analysis of SAMHD1 in HCT116 cells pretreated with or without P5091 (20 μM) and stimulated with and without cisplatin (5 μM) for 1 h.

**Fig. 5:**
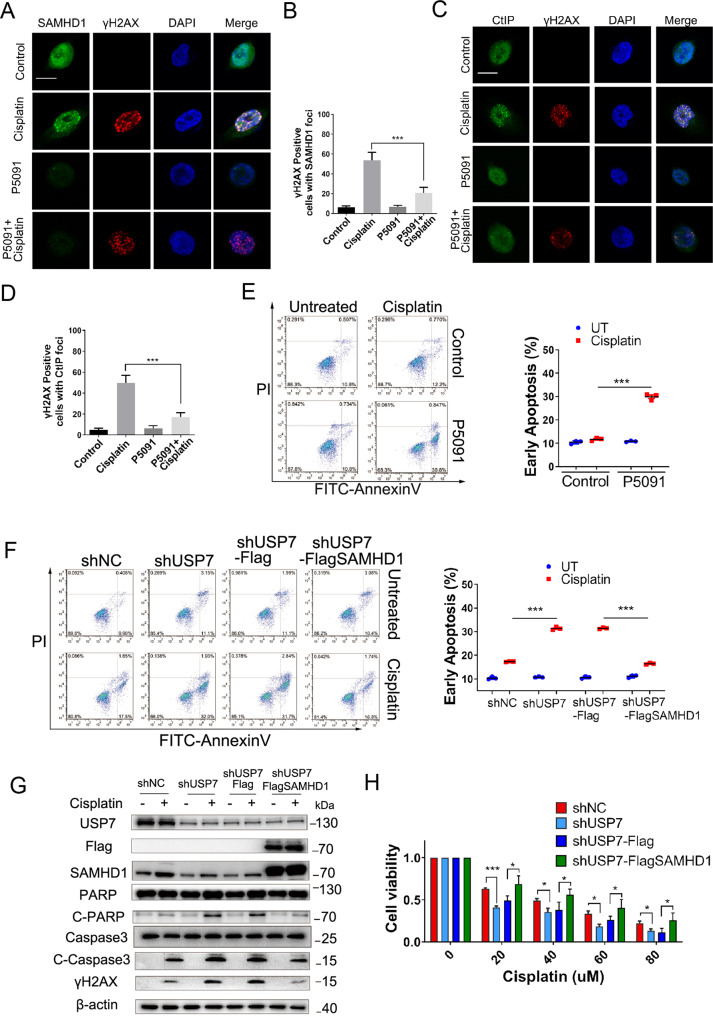
The USP7-SAMHD1 axis modulates cell survival and apoptosis under genotoxic insults. **A-D** HCT116 cells with or without P5091 (20 μM) pretreatment were stimulated with or without cisplatin (5 μM) for 4 h, and then stained with the anti-SAMHD1 or anti-CtIP and anti-γH2AX antibodies, DAPI, respectively, for immunofluorescence analysis. Scale bar, 10 μm. **E** HCT116 cells with or without P5091 (20 μM) administration were treated with cisplatin (10 μM) for 24 h followed by staining with PI and FITC-Annexin V, and analyzed by FACS; ***P < 0.001. **F** HCT116 shNC and shUSP7 cells or shUSP7-Flag and shUSP7-Flag-SAMHD1 cells were treated with cisplatin (20 μM) for 24 h followed by staining with PI and FITC-Annexin V, and analyzed by FACS; ***P < 0.001. **G** HCT116 shNC and shUSP7 cells or shUSP7-Flag and shUSP7-Flag-SAMHD1 cells were treated with cisplatin (5 μM) for 12 h followed by western blot analysis of USP7, Flag, SAMHD1, PARP, Cleaved-PARP, Caspase 3, Cleaved-Caspase 3, γH2AX. **H** HCT116 shNC and shUSP7 cells or shUSP7-Flag and shUSP7-Flag-SAMHD1 cells were treated with cisplatin at different concentrations for 24 h. Cell viability was assessed by CCK8 assay.

It has been recently reported that SAMHD1 participates in DNA damage repair. This has led us to explore the effect of SAMHD1 on tumor cell survival under genotoxic insults. Consequently, stable knockdown of SAMHD1 significantly reduces the survival rate of HCT116 and SW480 cells in a cisplatin and doxorubicin concentration-dependent manner, respectively (Figs. 4A, 5C; Fig. S3A). In contrast, stable overexpression Flag-SAMHD1 in shSAMHD1 colon cancer cells increases the survival rate in a cisplatin and doxorubicin concentration-dependent manner, respectively (Fig. 4B, D; Fig. S3B). Moreover, SAMHD1 promotes cell survival in a cisplatin treatment time-dependent manner under genotoxic insults in HCT116 and SW480 cells, respectively (Fig. S3C–F).

We next investigated the effect of SAMHD1 on the apoptosis of tumor cells induced by genotoxic insults. Under the treatment of cisplatin, shSAMHD1 cells demonstrated significantly increased apoptosis when compared to shNC cells, while stably expressing Flag-SAMHD1 cells significantly decreased apoptosis when compared to vector cells stably expressing Flag in HCT116 and SW480 cells, respectively (Figs. 4E, F; Fig. S3G, H). To elucidate whether the inhibitory effect of SAMHD1 on cell apoptosis depends on its ability of dNTP hydrolase, we stably expressed H206A/H207A SAMHD1 with no dNTP hydrolase activity in shSAMHD1 HCT116 and SW480 cells, respectively. Under the treatment of cisplatin, SAMHD1 wild-type and SAMHD1 AA both suppressed cell apoptosis in HCT116 and SW480 cells, respectively, with no statistical difference between their inhibitory effects, suggesting that SAMHD1 inhibition of cell apoptosis does not depend on the dNTP hydrolase activity (Fig. 4G). This notion was further confirmed by western blot analysis, in which significant increased apoptotic markers cleaved-PARP and cleaved-Caspase 3, and DSB marker γH2AX were observed in shSAMDH1 cells under the treatment of cisplatin, when compared to shNC cells. In addition, stably expressing Flag-SAMHD1 in shSAMHD1 colon cancer cells significantly decreased cleaved-PARP, cleaved-Caspase 3 and γH2AX when compared to Flag vector (Fig. 4H). Moreover, cisplatin induced formation of SAMHD1 and CtIP foci and their co-localization with γH2AX, respectively (Fig. S3I; J).

### Genotoxic insults or ROS induces deubiquitination and increase of SAMHD1 for DNA damage repair

It is widely known that chemotherapeutics elevate intracellular ROS levels, which contributes to their genotoxicity [29, 30]. We have thereby further verified that cisplatin and doxorubicin increase SAMHD1 protein expression without altering its mRNA level (Fig. 4I, J, Fig. S4H, I). Moreover, these agents induce the deubiquitination of SAMHD1 protein (Fig. 4K, S4J) and promote the interaction of SAMHD1 and CtIP (Fig. S4M, N).

The occurrence and development of tumors are accompanied by the up-regulation of ROS and the accumulation of DNA damage, and ROS can induce DNA damage and activate the DNA damage response signaling [31, 32]. We found that oxidative stress generated by H_2_O_2_ elevated SAMHD1 in HCT116 cells (Fig. S4A), HEK293 cells (Fig S4C), and H1299 (Fig. S4E), thus confirming the change of SAMHD1 in response to oxidative stress. In addition, N-acetylcysteine (NAC), a scavenger of free radicals, significantly inhibited the SAMHD1 increase induced by H_2_O_2_ (Fig. S4B, Fig. S4D, F). Quantitative PCR analysis revealed unchanged mRNA levels of SAMHD1 upon ROS stimulation (Fig. S4G), suggesting that post-translational modifications rather than transcriptional regulation is responsible for SAMHD1 protein overexpression. Indeed, the ubiquitination of SAMHD1 was significantly decreased under the stimulation of ROS (Fig. S4K, Fig. S4L), thus further supporting the importance of deubiquitination in upregulating SAMHD1 protein under oxidative stress. Previous studies have suggested that SAMHD1 promotes DNA end resection to facilitate DNA double-strand break repair by interacting with CtIP. In the current study, we have demonstrated that ROS promotes the interaction of SAMHD1 and CtIP (Fig. S4O–Q). This is in keeping with the observations that H_2_O_2_ induces formation of SAMHD1 and CtIP foci as well as their co-localization with DNA damage marker γH2AX, respectively (Fig. S3K, Fig. S3L). In addition, the co-localization between SAMHD1 and CtIP upon cisplatin treatment was also observed (Fig. S3M).

### The USP7-SAMHD1 axis interactions facilitate cell survival and reduce apoptosis under genotoxic insults

To further elucidate the regulation of USP7 on SAMHD1 protein stability, we next explored the effect of USP7-SAMHD1 axis on tumor cell survival under genotoxic insults. First, cisplatin, doxorubicin, H_2_O_2_ was found to promote the binding of USP7 and SAMHD1 (Fig. 4L; Fig. S5A–C). We found that the stabilization of SAMHD1 by USP7 were heightened in the presence of DNA damage agents (Figure S1I). Besides, the decrease of SAMHD1 ubiquitination by USP7 were also heightened in response to DNA damage agents (Figure S1R). The co-localization between SAMHD1 and USP7 upon cisplatin treatment was also observed (Fig. 4M). Furthermore, the USP7 inhibitor P5091 suppresses the increase of SAMHD1 protein induced by cisplatin or doxorubicin (Fig. 4N; Fig. S5D). In addition, the formation of SAMHD1 foci and co-localization with DNA damage marker γH2AX induced by cisplatin was significantly inhibited by P5091 (Fig. 5A, B). Similar findings were obtained with respect to the downstream CtIP foci and co-localization with γH2AX (Fig. 5C, D), suggesting that USP7-SAMHD1-CtIP axis contributes to DDR induced by chemotherapeutic agents.

As for cell apoptosis, shUSP7 cells demonstrated significantly increased apoptosis when compared to shNC cells under the treatment of cisplatin or doxorubicin, whereas stably expressing Flag-SAMHD1 cells in shUSP7 cells showed significantly decreased apoptosis when compared to the HCT116 cells stably expressing the Flag vector (Fig. 5F; Fig. S5G, H). Stable knockdown of USP7 significantly reduced the survival rate of HCT116 cells in a cisplatin and doxorubicin concentration-dependent manner. Conversely, stable overexpression of Flag-SAMHD1 in shUSP7 HCT116 cells increased the survival rate (Fig. 5H; Fig. S5E, F). P5091 treatment significantly increased apoptosis induced by cisplatin or doxorubicin (Fig. 5E; Fig. S5I). The results were further confirmed by western blot analysis, in which apoptotic markers cleaved-PARP and cleaved-Caspase 3 and DSB marker γH2AX were significantly increased in shUSP7 HCT116 cells when compared to shNC cells under treatment of cisplatin. In addition, stably expressing Flag-SAMHD1 in shUSP7 HCT116 cells significantly decreased cleaved-PARP, cleaved-Caspase 3 and γH2AX when compared to cells expressing Flag vector (Fig. 5G).

Furthermore, HCT116 cells transfected with USP7 WT-SAMHD1 WT demonstrated increased survival rate compared to USP7 C223S-SAMHD1 WT, USP7 WT- SAMHD1 K421R or USP7 C223S-SAMHD1 K421R in a cisplatin time-dependent manner (Fig S5J). Similarly, USP7 WT-SAMHD1 WT demonstrated reduced cell apoptosis rate compared to other groups (Fig S5K). Both SAMHD1 WT and SAMHD1 K421R could reduce cell apoptosis rate compared to Flag vector in HCT116 shUSP7 cells (Fig S5L). SAMHD1 WT and H206A/H207A SAMHD1 both increased the survival rate under cisplatin treatment compared to Flag in shUSP7 HCT116 cells with no significant difference (Fig S5M).

### SAMHD1 and USP7 are highly expressed in multiple cancer tissues

**Fig. 6:**
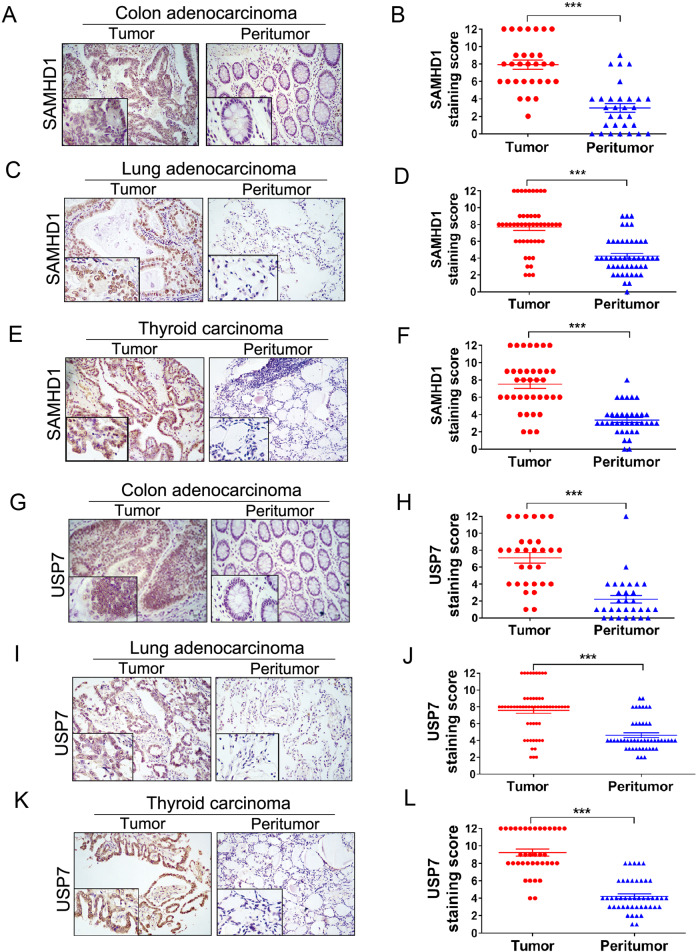
SAMHD1 and USP7 are highly expressed in cancer tissues. **A–F** Representative sections and semiquantitative analyses of SAMHD1 expression in early-stage (stage I and II) colonic adenocarcinoma (**A** and **B**, *n* = 30), lung adenocarcinoma (**C** and **D**, *n* = 48), and thyroid carcinoma (**E** and **F**, *n* = 58). Data are expressed as mean ± SEM. ***P < 0.001 (Mann-Whitney test). **G-L** Representative sections and semiquantitative analyses of USP7 expression in colonic adenocarcinoma (**G** and **H**, *n* = 30), lung adenocarcinoma (**I** and **J**, *n* = 48), and thyroid carcinoma (**K** and **L**, *n* = 58). Data are expressed as mean ± SEM. ***P < 0.001 (Mann-Whitney test).

A number of previous studies have indicated the implication of SAMHD1 in the initiation and development of cancer, while inconsistent findings have been obtained in different tumor types [33]. To explore the role of SAMHD1 in tumorigenesis, we first examined the SAMHD1 expression by immunohistochemistry in several early-stage (stages I and II) human cancers including colonic and lung adenocarcinomas as well as thyroid carcinoma. To that end, semiquantitative immunohistochemical analysis revealed that SAMHD1 was highly expressed in carcinomas when compared to their paired peritumor tissues, suggesting that SAMHD1 might be implicated in tumor progression (Fig. 6A–F; Fig. S6A–C). Moreover, significantly higher levels of USP7 were found in colonic and lung adenocarcinomas as well as thyroid carcinoma when compared to their paired peritumoral tissues (Fig. 6G–L; Fig. S6D–F).

### Inhibition of the USP7-SAMHD1 axis improves chemosensitivity

**Fig. 7:**
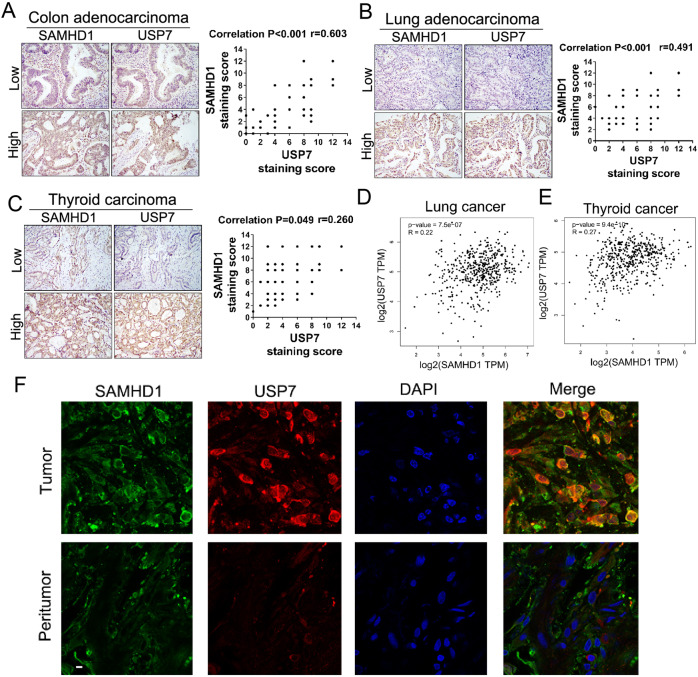
USP7 and SAMHD1 are positively correlated in various cancer types. Expressions of USP7 and SAMHD1 are positively correlated in consecutive sections of colonic carcinoma (**A**), lung adenocarcinoma (**B**) and thyroid carcinoma tissues (**C**). Results of spearman correlation analyses of P value and correlation coefficient are shown. **D**, **E** Correlation of USP7 and SAMHD1 expressions in human lung and thyroid carcinomas in the GEPIA database. **F** Colonic adenocarcinoma and peritumoral tissues stained with the anti-SAMHD1 antibody, anti-USP7 antibody, and DAPI, respectively, for immunofluorescent analyses. Scale bar, 10 μm.

To further explore the role of USP7-SAMHD1 in human cancers, semiquantitative immunohistochemical analyses for USP7 and SAMHD1 were performed in tumorous tissues. A significant positive correlation between USP7 and SAMHD1 expression was identified in all three tumor types, suggesting that the existence of the USP7-SAMHD1 axis in various human cancers (Fig. 7A–C). Furthermore, data from The Cancer Genome Atlas (TCGA) also indicate a positive correlation between USP7 and SAMHD1 expressions in various cancer types (Figs. 7D, E; Fig. S7E–L). In addition, co-localization of USP7 and SAMHD1 was identified in colonic adenocarcinoma tissues while this phenomenon was only rarely observed in peritumoral tissues (Fig. 7F).

**Fig. 8:**
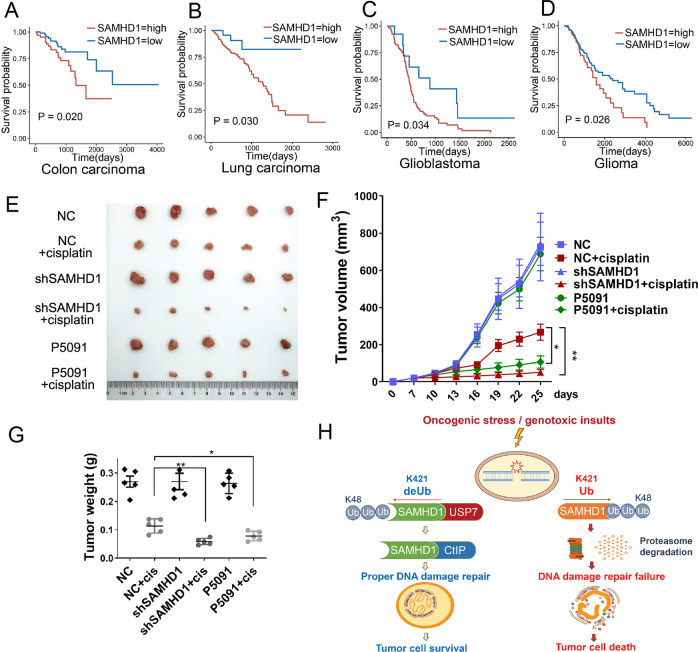
Inhibition of the USP7-SAMHD1 axis improves chemosensitivity. **A–D** A higher SAMHD1 expression is significantly associated with an unfavorable survival outcome in patients with carcinoma of various organs who received chemotherapy according to The Cancer Genome Atlas (TCGA) data. **E–G** Tumor growth assay in nude mice subcutaneously inoculated with shNC or shSAMHD1 cells. The mice were treated with cisplatin, with or without P5091. The images of tumors were acquired (**E**), and their volume (**F**) or weight (**G**) were determined. Data are shown as mean ± SD (*n* = 5 for each group). *P < 0.05; **P < 0.01. **H** A schematic model showing the proposed role of the USP7-SAMHD1 axis in controlling tumor cell survival and chemosensitivity.

We then analyzed the potential prognostic significance of SAMHD1 expression in patients with various cancers who received chemotherapy according to TCGA data. To that end, a higher SAMHD1 expression is significantly associated with an unfavorable survival outcome in patients with colonic adenocarcinoma (Hazard ratio [HR] = 2.15, *P* = 0.020), lung adenocarcinoma (HR = 3.34, *P* = 0.030), and glioblastoma (HR = 1.99, *P* = 0.034) and glioma (HR = 1.56, *P* = 0.026), suggesting that SAMHD1 may contribute to chemotherapy insensitivity in these cancers (Fig. 8A–D; Fig. S7A–D). Prognostic analysis of USP7 expression in patients with various cancers who received chemotherapy according to TCGA data demonstrated no significant association with survival outcome (*P* > 0.05)(Fig S7M–P), which might be attributed to the various substrates and multiple functions for USP7. Using a nude mice model, we further explored the effect of the USP7-SAMDH1 axis on chemosensitivity. We injected stable SAMHD1 knockdown (shSAMHD1) or control (shNC) HCT116 cells subcutaneously into the nude mice. The shNC mice were randomly divided into groups administrated intraperitoneally with vehicle, cisplatin, P5091, or P5091 together with cisplatin twice a week; while the shSAMHD1 mice were divided into groups treated with vehicle or cisplatin twice a week. Consequently, the results showed that shSAMHD1 cells were more sensitive to cisplatin when compared to shNC cells as determined by tumor volume (Fig. 8E, F) and weight (Fig. 8G). Importantly, administration of P5091 sensitized HCT116 cells to cisplatin (Fig. 8E–G). Taken together, the findings suggest that SAMHD1 may be a potential prognostic biomarker in the adjuvant and neoadjuvant setting, and that the combination of cytotoxic chemotherapy and USP7 inhibition might improve chemotherapy sensitivity in various cancers (Fig. 8H).

## Discussion

The observations in this study have demonstrated that SAMHD1 is associated with and stabilized by the deubiquitinase USP7 at K421. Further, SAMHD1 is upregulated in various early-stage human carcinomas and positively correlated with USP7. Furthermore, USP7-stabilized SAMHD1 promotes DDR by interacting with DSB initiator CtIP, thus resulting in DNA damage repair induced by ROS and genotoxic insults. Importantly, USP7 inhibitor sensitizes cancer cells to chemotherapy by decreasing SAMHD1, suggesting that SAMHD1 stabilization by USP7 promotes DNA damage repair to overcome oncogenic stress and affect chemotherapy sensitivity.

Tumorigenesis is accompanied by up-regulation of ROS and accumulation of DNA damage, and the former can induce DNA damage and activate the DNA damage repair signaling [34, 35]. SAMHD1 reportedly promotes DNA end resection to facilitate DNA DSB repair by interacting with CtIP independent of its dNTP hydrolysis ability [12]. In addition, SAMHD1 facilitates degradation of nascent DNA at stalled replication forks in the replication stress response [36]. Our findings in human cancerous tissues suggest that SAMHD1 might be implicated in early-stage carcinomas to overcome elevated DNA damage and oncogenic stress. In eukaryotic cells, ubiquitination plays an essential role in the assembly as well as disassembly of DDR factors at break sites [37, 38]. Both ROS and genotoxic insults, such as cisplatin or doxorubicin, may increase SAMHD1 by inducing its protein deubiquitination and promote the interaction of SAMHD1 with CtIP for DDR. These findings have indicated a novel deubiqutination regulation of SAMHD1 during tumorigenesis.

SAMHD1 has been known as an effector in innate immunity, a restrictor for retroviruses, and a regulator in the cell cycle, which largely depends on its ubiquitination regulatory mechanism [39]. To escape restriction by SAMHD1, HIV-2 and SIV viruses evolved viron-associated Vpx protein degrades SAMHD1 through proteasome pathway via hijacking the Cul4A/DDB1/DCAF1 E3 ligase [40]. It has been recently reported that TRIM21 is the E3 ubiquitin ligase of SAMHD1 for its degradation in enterovirus 71 (EV71) infection [27]. However, the specific deubiquitinase of SAMHD1 still remains unclear. In this study, we have found for the first time that USP7, a pivotal deubiquitinase, directly interacts with the HD domain of SAMHD1 and stabilizes SAMHD1 from degradation through the proteasomal pathway. Colocalization of USP7 and SAMHD1 has been further confirmed by confocal microscopy. Specifically, USP7 increases expression and prolongs the half-life of SAMHD1 protein by reducing its K48-linked polyubiquitination at K421. K421 is the predominant site of SAMHD1 polyubiquitination for degradation. In addition, K492 site of SAMHD1 is not the predominant site that USP7 deubiquitinate SAMHD1, which might participate in biological processes other than protein degradation. These findings provide further insight into potential specific regulation of SAMHD1 dysfunction.

A variety of chemotherapeutic agents induce DNA damage to kill tumor cells, while overexuberant DDR contributes to chemoresistance and worse prognosis in various tumor types [41, 42]. The involvement of SAMHD1 in DNA damage repair suggests a potential role of SAMHD1 in regulating chemosensitivity. Indeed, SAMHD1 knockdown significantly increases chemosensitivity to cisplatin and doxorubicin in tumor cells. Multiple chemotherapeutic agents induce DNA damage and apoptosis in cancer cells [43, 44]. Under the treatment of chemotherapy drugs, SAMHD1 significantly inhibits apoptosis independent of its dNTP hydrolase activity. Several previous studies have obtained similar results in this regard. For example, targeting SAMHD1 by the Vpx protein has been found to benefit cytarabine therapy for hematological malignancies [45]. Moreover, SAMHD1 expression levels determine acute lymphoblastic leukemia cell response to nelarabine [46]. Therefore, these findings, along with our observations, suggest that SAMHD1 is a potential therapeutic target for improving chemotherapy efficacy in treating malignancies.

Emerging evidence has suggested the implication of USP7 in initiation and development of cancer [47–49]. For instance, USP7 deubiquitinates and stabilizes N-myc protein to promote neuroblastoma progression [50]. In addition, USP7 deubiquitinates β-catenin and activate Wnt signaling to promote colon cancer development [51]. We found that the USP7-SAMHD1 axis contribute to the DDR and chemotherapy insensitivity of cancer cells. Importantly, inhibition of USP7 suppresses chemotherapeutic agents-induced SAMHD1 expression and prevents the formation of SAMHD1 or CtIP foci and their co-localization with γH2AX. Further, activation of the USP7-SAMHD1 axis reduces chemosensitivity and inhibits apoptosis. Attesting to function, USP7 was highly expressed in a variety of tumoral tissues and positively correlated with the SAMHD1 expression. Moreover, the patients with high SAMHD1-expressing tumors who received chemotherapy had unfavorable survival outcomes. Administration of USP7 inhibitor P5091 (25 mg/kg) has been found to decrease the growth rate of tumor in vivo. In this study, we used relatively low concentration of P5091 (5 mg/kg) to treat the tumor in vivo. As a result, the tumor volume and weight showed no significant change after P5091 treatment alone. But the tumor volume and weight were decreased in “P5091 and cisplatin” group. We further demonstrated that blockade of USP7 by its specific inhibitor P5091 sensitizes cancer cells to cisplatin using a nude mice model, thus further indicating its potential role in chemotherapy sensitivity.

In summary, we have identified a deubiquitination-dependent regulatory mechanism of the USP7-SAMHD1 axis for sustaining DNA damage repair in tumorigenesis and cell survival under genotoxic insults. These findings suggest that SAMHD1 may be a potential biomarker for chemosensitivity, and that combination of chemotherapy and USP7 inhibition may potentially help improve chemotherapy sensitivity in various cancer types.

## Methods

### Cell culture

HCT116, SW480, H1299, HEK293, Hela, A549, TPC-1 cells were purchased from Cell Bank in the Chinese Academy of Sciences Shanghai. HCT116, SW480, TPC-1, and H1299 were cultured in RPMI 1640 medium; HEK293, A549, and Hela were cultured in high-glucose Dulbecco’s modified Eagle’s medium (DMEM), supplemented with10% fetal bovine serum (FBS)(CLARK, Australia), penicillin (100U), and streptomycin (100 g/ml).

### Antibodies and reagents

Antibodies used in this study includeUSP7(Western blot: #4833, CST; ab4080, Abcam; Immunohistochemistry: NB100-513, NOVUS), SAMHD1 (Western blot:#12361, CST; IP: ab245389, Abcam; Immunohistochemistry/Immunofluorescence: ab128107, Abcam), CtIP (Western blot: #9201, CST;Immunofluorescence:NB100-79810), β-actin (AC004, ABclonal), Flag (SG4110-16, Shanghai Genomics Technology), Myc (SG4110-18, Shanghai Genomics Technology), HA (#3724, CST), Ubiquitin (#3933S, CST), PARP (#9532, CST),Cleaved PARP (#5625, CST), Caspase3 (#9662, CST), Cleaved Caspase3 (#9664, CST), Phospho-Histone H2A.X (Ser139) (#9718, CST; #80312, CST).

CHX (S7418), P5091 (S7132), Cisplatin (S1166), Doxorubicin (Adriamycin) HCl (S1208), and MG132 (S2619) were purchased from Selleck. PI (propidium iodide, ST511) and NAC (S0077) were from Beyotime. IPTG (I6758) were from Sigma-Aldrich.

### Plasmid constructions, transfection, and lentivirus infection

SAMHD1 and USP7 expression plasmids were purchased from Shanghai GeneChem Company. Mutagenesis (SAMHD1 K421R, SAMHD1 K492R, SAMHD1 K494R, SAMHD1 H206A/D207A, and USP7C223S) was performed based on Quick Change Site-Directed Mutagenesis Kit (Stratagene, La Jolla, CA). Specific siRNAs for USP7 were purchased from RiboBio Co., Ltd., Guangzhou, China. The plasmids were confirmed by sequencing and transfected into HEK293, HCT116 or SW480 cells using Lipofectamine 3000 reagent (Thermo Fisher Scientific, USA) according to the manufacturer’s instructions. Cells were harvested 48 hours after transfection. For lentiviral production and infection, control shRNA (shNC) lentivirus, shRNA against SAMHD1 (shSAMHD1) lentivirus, shRNA against USP7(shUSP7) lentivirus, Flag-vector overexpression lentivirus (Flag) and SAMHD1 overexpression lentivirus (Flag-SAMHD1) were purchased from Shanghai GeneChem Company. Stably infected cell lines were selected by puromycin after lentivirus infection for 5 days.

### Mass spectrometry

We overexpressed Flag-SAMHD1 in HEK-293 cells and collected the cells after 48 hours. IP lysis was used for cell lysis, and Flag-beads was added to the protein lysis solution. The solution was mixed overnight in a chromatography cabinet at 4 °C, which was centrifuged and washed with PBS at 4 °C for 3 times. The beads were boiled with 2×loading in a water bath for 10 minutes. The immunoprecipitated protein were subjected to western blot and excised from the gel for digestion and mass spectrometry. Interaction protein and peptide identifications were conducted by database search.

### Western blot analysis and Co-immunoprecipitation (Co-IP)

Cells were lysed for 30 min on ice with IP lysis buffer supplemented with protease inhibitor cocktails, then the lysed protein was harvested by centrifugation at 15,000 rpm for 20 min at 4 °C. Protein concentration was assessed by G250 and 40 μg of cell lysate were adopted for samples. Protein samples were separated on 8%, 10%, or 12% SDS PAGE and transferred to PVDF membrane (Millipore, IPVH00010) for two hours at 80 V. After block in 5% BSA in TBST for one hour at room temperature, the membranes were probed with specific primary antibodies at 4 °C overnight. The membranes were then washed with TBST three times followed by incubation with HRP-conjugated secondary antibody at room temperature for two hours. After three washes, bands were detected by enhanced chemi-luminescence detection kit (Thermo Fisher Scientific, 32106) and visualized via the DNR western blot detection system.

Cells were lysed with IP lysis buffer (25 mM Tris, pH 7.6, 150 mM NaCl, 1%Nonidet P-40, 1 mM EDTA), and 1 mg protein was incubated with antibody and protein A/G-Sepharose (Santa Cruz, sc-2003) on a rocking platform overnight at 4 °C. Next, the beads were harvested by centrifugation for 5 min at 700 g at 4 °C and the supernatant was removed. The beads were then resuspended in IP lysis buffer and repeatedly inverted on a rocking platform for 10 min. This wash was repeated three times to remove the nonspecific binding protein and obtain purified protein complexes that are bound to the antibody-coated beads. Finally, the beads were resuspended with loading buffer for Western blot analysis.

### GST-pulldown

The bacterial expression constructs (pGEX-4T-1) containing the target genes (SAMHD1 or USP7) were transformed into BL21-competent cells (Takara). Cells were induced to overexpress the GST-fusion protein by 1 mM IPTG for 3 h while shaking at 30 °C. Cells were resuspended in bacterial lysis (PBS containing 1 mM PMSF, 5 mM β-mercaptoethanol,0.5% TritonX-100, and 2 mM EDTA), followed by ultrasonication. The proteins were purified by a single step using glutathione bead according to the manufacturer’s protocol (Promega Science). In vitro transcription and translation of SAMHD1 or USP7 proteins were performed by T7-TNT Kits (Promega, L1170) in accordance with the manufacturer’s instructions. GST pulldown assays were performed as previously described [52].

### Immunofluorescent analysis

For immunofluorescence in cultured cells, the culture medium was discarded and washed 4 times with PBS, then subjected to treatment with tissue fixative for 20 minutes. The cells were then washed 3 times with PBS and treated by 0.25% TritonX-100 for 15 minutes. The samples were washed 3 times with PBS before block with 5% BSA for 1 hour, and then incubated the primary antibody overnight at 4 °C. After three times wash with PBS the next day, the corresponding fluorescent secondary antibody (1:400) was incubated at room temperature for 1 h in the dark. After three times wash with PBS, the samples were stained with DAPI for 5 minutes and mount upside down on the glass slide, which was protected from light and dry for observation under the laser confocal microscope in a dark room.

For tissue immunofluorescence, the colonic tissues were fixed in 4% paraformaldehyde overnight and then sections (100 µm) were incubated in 5% normal donkey serum in PBS containing 0.5% Triton X-100 to block nonspecific binding for 1 h at room temperature. Primary antibody against USP7 and SAMHD1 were diluted in PBS containing 0.1% Triton X-100 and 1% donkey serum and incubated with sections overnight at 4 °C. The tissue sections were subjected to anti-Rabbit-Cy2 or anti-mouse-Cy2 secondary antibodies (1:250, Jackson), followed by DAPI staining. Then tissue sections were then mounted and observed using a confocal laser scanning microscopy (Zeiss LSM880).

### RNA isolation and quantitative real-time PCR

Total RNA was isolated from cells using Trizol reagent. Reverse transcription was performed using a PrimeScript RT Reagent Kit (Takara, RR037A), according to the manufacturer’s instructions. The cDNA was quantified by quantitative real-time PCR with SYBR®Premix Ex Taq™ II (Takara, RR820A) on Mx3000P instrument (Agilent StrataGene). The sequences of SAMHD1 primers were Forward: TGCAGAGCAGCTGATTCGAG; Reverse: ATAACATCGCCATCCTGCGG. SAMHD1 mRNA expression was calculated relative to expression of the housekeeping gene β-actin using Stratagene Mx3000P software.

### Cell proliferation assay

HCT116 or SW480 cells were seeded in triplicate at a density of 1 × 10^4^cells per well into 96-well plates. After 24-hour incubation in complete RPMI 1640 with 10% FBS, cells were exposed to Cisplatin or Doxorubicin at different concentrations. Upon measurement, RPMI 1640 medium and CCK8(Cell Counting Kit-8) staining solution was added to cells at each well for 2 hours at 37 °C. The absorbance was measured at 450 nm daily using an absorbance reader (TECAN, Switzerland). The percentage of cell survival was then calculated.

### Flow cytometric analysis

To investigate cell apoptosis, HCT116 or SW480 cells were treated with Cisplatin for 24 h, followed by incubation with PI and FITC-Annexin V (BD Phamingen, 556547). In addition, HCT116 cells were treated with Doxorubicin for 24 h, followed by incubation with APC and 7-AAD (KeyGEN BioTECH, KGA1026). The percentage of apoptotic cells was then measured according to the manufacturer’s protocol.

### Immunohistochemistry

The tissue microarrays of early stage of colon adenocarcinoma (HCol-Ade060CS1-01), lung adenocarcinoma (HLugA150CS03), thyroid carcinoma (HThyP120CS02) were purchased from Shanghai Outdo Biotech Company, China. After deparaffinizing in xylene and rehydrating in graded ethanol, tissue microarrays were immersed in citrate buffer for antigen retrieval. Endogenous peroxidase was quenched using 3% hydrogen peroxide for 30 min. To decrease the nonspecific staining, 10% normal goat serum was subsequently used to block tissue collagen for 30 min. Tissue sections were then incubated with antibody anti-SAMHD1 (ab128107, 1:2000, Abcam) or USP7 (NB100-513, 1:1000, NOVUS) for 60 minutes at room temperature (24–27 °C). After that, biotinylated secondary antibody and streptavidin-biotin peroxidase were used to incubate tissue sections for 10 min each in turn. Slides were stained with DAB chromogenic reagent for 60 s, afterwards counterstained with hematoxylin. UltraSensitive^TM^ SPIHC Kit(KIT-9720, Maixin Inc., Fujian, China) were used in this experiment.

The stained sections were reviewed and scored by two investigators independently who were blinded to the clinical information. We adopted a semi-quantitative scoring method to assess the expression of SAMHD1 and USP7. The staining intensity was divided into 0 (no staining), 1 (weak staining), 2 (moderate), and 3 (strong). The percentage of cells stained was categorized as 0 (0–5%), 1 (6–25%), 2 (26–50%), 3 (51–75), and 4 (76–100%). The final scores were generated by multiplying the staining intensity by percentage of cells, which were classified as: 0-4, low; 5–8, medium; 9–12, high; respectively.

### Tumor xenografts

We use the nude mouse model to further analyze the role of USP7-SAMHD1 axis in colon cancer chemosensitivity. A total of 48 female nude mice (BALB/cA-nu Mice) at 4 weeks of age were subcutaneously inoculated 5 × 10^6^ HCT116 shNC and HCT116 shSAMHD1 cells. After 1 week, the mice were randomly divided into shNC, shNC+cisplatin, shSAMHD1, shSAMHD1+cisplatin, P5091, and P5091 + cisplatin groups, respectively. Cisplatin and P5091 were administered twice a week at a concentration of 2 mg/kg and 5 mg/kg, respectively. The tumor size was measured daily. The tumor volume was calculated (length × width^2^). The mice were sacrificed after 25 days, and the tumors were removed for subsequent analyses. All animal experiments were approved by the Institutional Animal Care and Use Committee of China Medical University.

### Statistical analysis

SPSS version 20.0 (SPSS Inc., Chicago IL, USA) was used for statistical analyses. Two-tailed Student’s *t*-test was used for continuous variables. Spearman’s rank correlation test was used to evaluate the correlation between SAMHD1 and USP7 expression. Survival outcomes were assessed by the Kaplan-Meier method, and the log-rank test was used to compare the differences between the groups. In addition, patients with carcinoma of various organs who received chemotherapy from The Cancer Genome Atlas (TCGA) data were analyzed. The Cox proportional hazards model was used to investigate the association of survival time and SAMHD1 expression. A *P* < 0.05 was considered statistically significant.

## Supplementary information

Supplementary Figures

Supplementary Figure Legends

## Data Availability

All data generated or analyzed during this study are included in this article and its Supplementary Information Files.
