# Supplementary material for: De-ubiquitination of SAMHD1 by USP7 promotes DNA damage repair to overcome oncogenic stress and affect chemotherapy sensitivity
Source: Oncogene. 2023 Apr 20;42(22):1843–56. doi: 10.1038/s41388-023-02667-w (PMC10238270; doi:10.1038/s41388-023-02667-w)

Figure S1

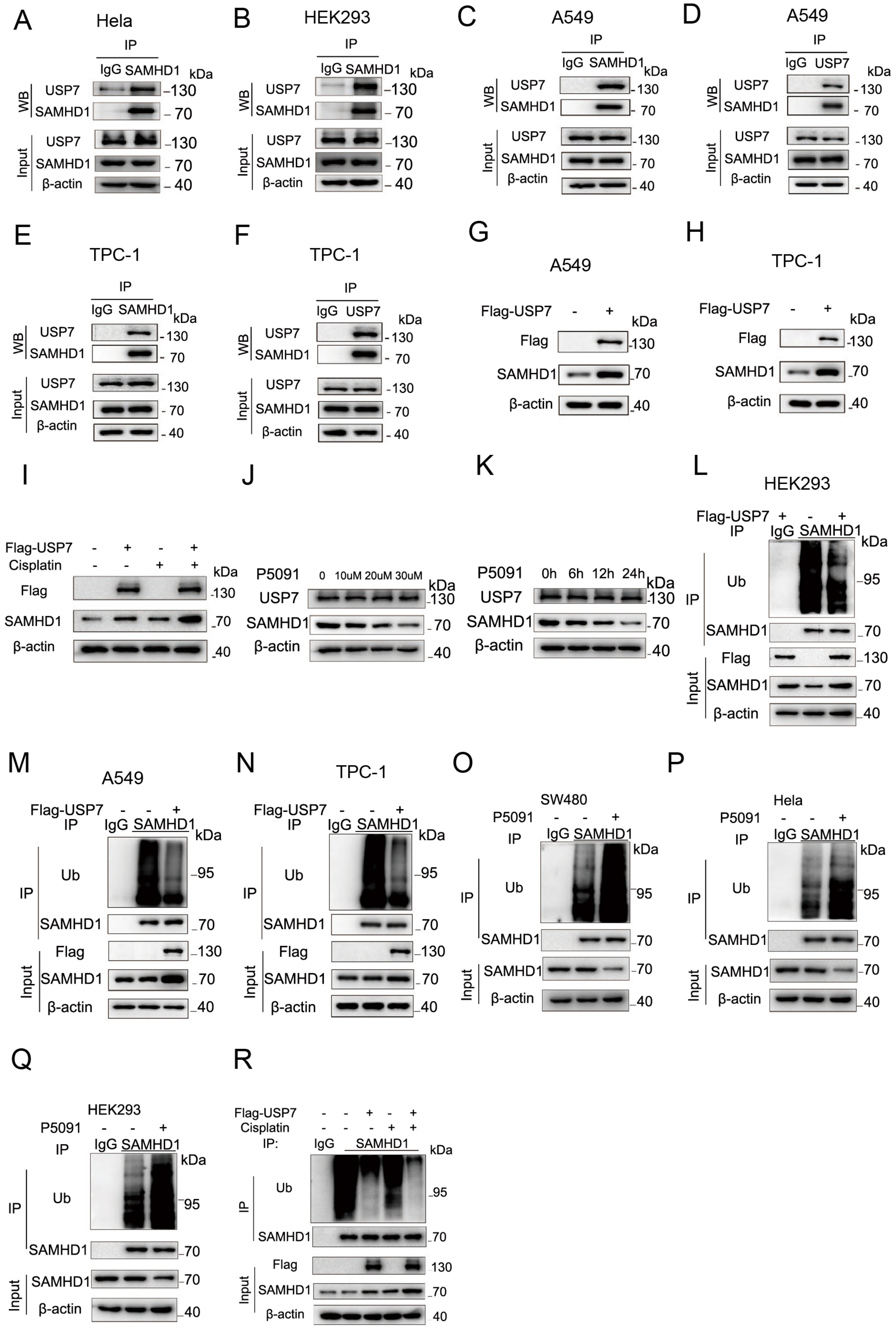
A
Hela
IP
IgG SAMHD1
kDa
WB
USP7
SAMHD1
Input
USP7
SAMHD1
β-actin
130
70
130
70
40
B
HEK293
IP
IgG SAMHD1
kDa
WB
USP7
SAMHD1
Input
USP7
SAMHD1
β-actin
130
70
130
70
40
C
A549
IP
IgG SAMHD1
kDa
WB
USP7
SAMHD1
Input
USP7
SAMHD1
β-actin
130
70
130
70
40
D
A549
IP
IgG USP7
kDa
WB
USP7
SAMHD1
Input
USP7
SAMHD1
β-actin
130
70
130
70
40
E
TPC-1
IP
IgG SAMHD1
kDa
WB
USP7
SAMHD1
Input
USP7
SAMHD1
β-actin
130
70
130
70
40
F
TPC-1
IP
IgG USP7
kDa
WB
USP7
SAMHD1
Input
USP7
SAMHD1
β-actin
130
70
130
70
40
G
A549
Flag-USP7 - +
kDa
Flag
SAMHD1
β-actin
130
70
40
H
TPC-1
Flag-USP7 - +
kDa
Flag
SAMHD1
β-actin
130
70
40
I
Flag-USP7 - + - +
Cisplatin - - + +
kDa
Flag
SAMHD1
β-actin
130
70
40
J
P5091 0 10uM 20uM 30uM
kDa
USP7
SAMHD1
β-actin
130
70
40
K
P5091 0h 6h 12h 24h
kDa
USP7
SAMHD1
β-actin
130
70
40
L
HEK293
Flag-USP7 + - +
IP IgG SAMHD1
kDa
IP
Ub
SAMHD1
Input
Flag
SAMHD1
β-actin
95
70
130
70
40
M
A549
Flag-USP7 - - +
IP IgG SAMHD1
kDa
IP
Ub
SAMHD1
Input
Flag
SAMHD1
β-actin
95
70
130
70
40
N
TPC-1
Flag-USP7 - - +
IP IgG SAMHD1
kDa
IP
Ub
SAMHD1
Input
Flag
SAMHD1
β-actin
95
70
130
70
40
O
SW480
P5091 - - +
IP IgG SAMHD1
kDa
IP
Ub
SAMHD1
Input
SAMHD1
β-actin
95
70
70
40
P
Hela
P5091 - - +
IP IgG SAMHD1
kDa
IP
Ub
SAMHD1
Input
SAMHD1
β-actin
95
70
70
40
Q
HEK293
P5091 - - +
IP IgG SAMHD1
kDa
IP
Ub
SAMHD1
Input
SAMHD1
β-actin
95
70
70
40
R
Flag-USP7 - - + - +
Cisplatin - - - + +
IP: IgG SAMHD1
kDa
IP
Ub
SAMHD1
Input
Flag
SAMHD1
β-actin
95
70
130
70
40

Figure S2

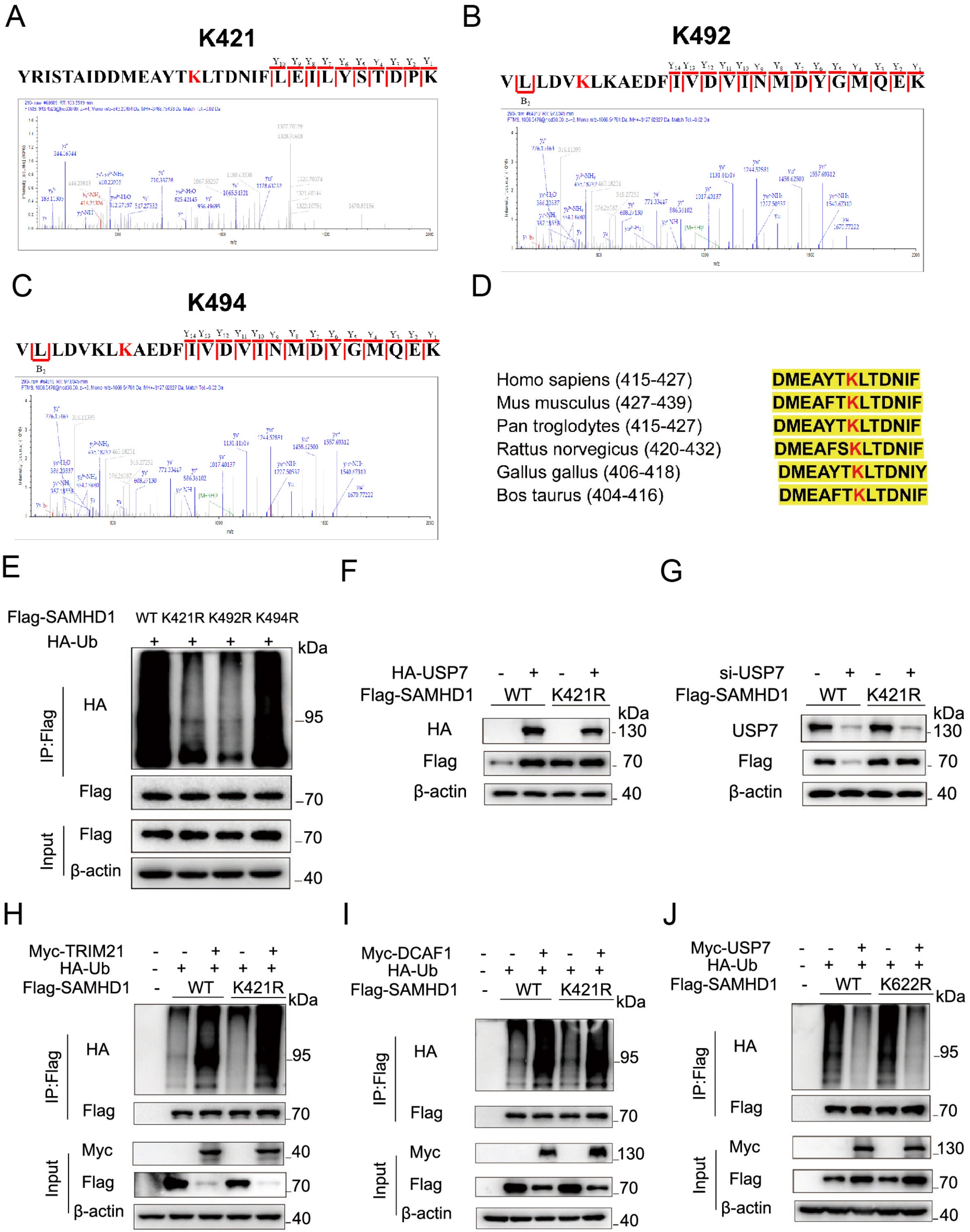

A
K421
YRISTAIDDMEAYTKLTDNIFLEILYSTDPK
B
K492
VLLDVKLKAEDFIVDVINMDYGMQEK
C
K494
VLLDVKLKAEDFIVDVINMDYGMQEK
D
Homo sapiens (415-427) DMEAYTKLTDNIF
Mus musculus (427-439) DMEAFTKLTDNIF
Pan troglodytes (415-427) DMEAYTKLTDNIF
Rattus norvegicus (420-432) DMEAFSKLTDNIF
Gallus gallus (406-418) DMEAYTKLTDNIY
Bos taurus (404-416) DMEAFTKLTDNIF
E
Flag-SAMHD1 WT K421R K492R K494R
HA-Ub + + + +
kDa
IP:Flag
HA
95
Flag
70
Input
Flag
70
β-actin
40
F
HA-USP7 - + - +
Flag-SAMHD1 WT K421R
kDa
HA 130
Flag 70
β-actin 40
G
si-USP7 - + - +
Flag-SAMHD1 WT K421R
kDa
USP7 130
Flag 70
β-actin 40
H
Myc-TRIM21 - - + - +
HA-Ub - + + + +
Flag-SAMHD1 - WT K421R
kDa
IP:Flag
HA
95
Flag
70
Input
Myc
40
Flag
70
β-actin
40
I
Myc-DCAF1 - - + - +
HA-Ub - + + + +
Flag-SAMHD1 - WT K421R
kDa
IP:Flag
HA
95
Flag
70
Input
Myc
130
Flag
70
β-actin
40
J
Myc-USP7 - - + - +
HA-Ub - + + + +
Flag-SAMHD1 - WT K622R
kDa
IP:Flag
HA
95
Flag
70
Input
Myc
130
Flag
70
β-actin
40

Figure S3

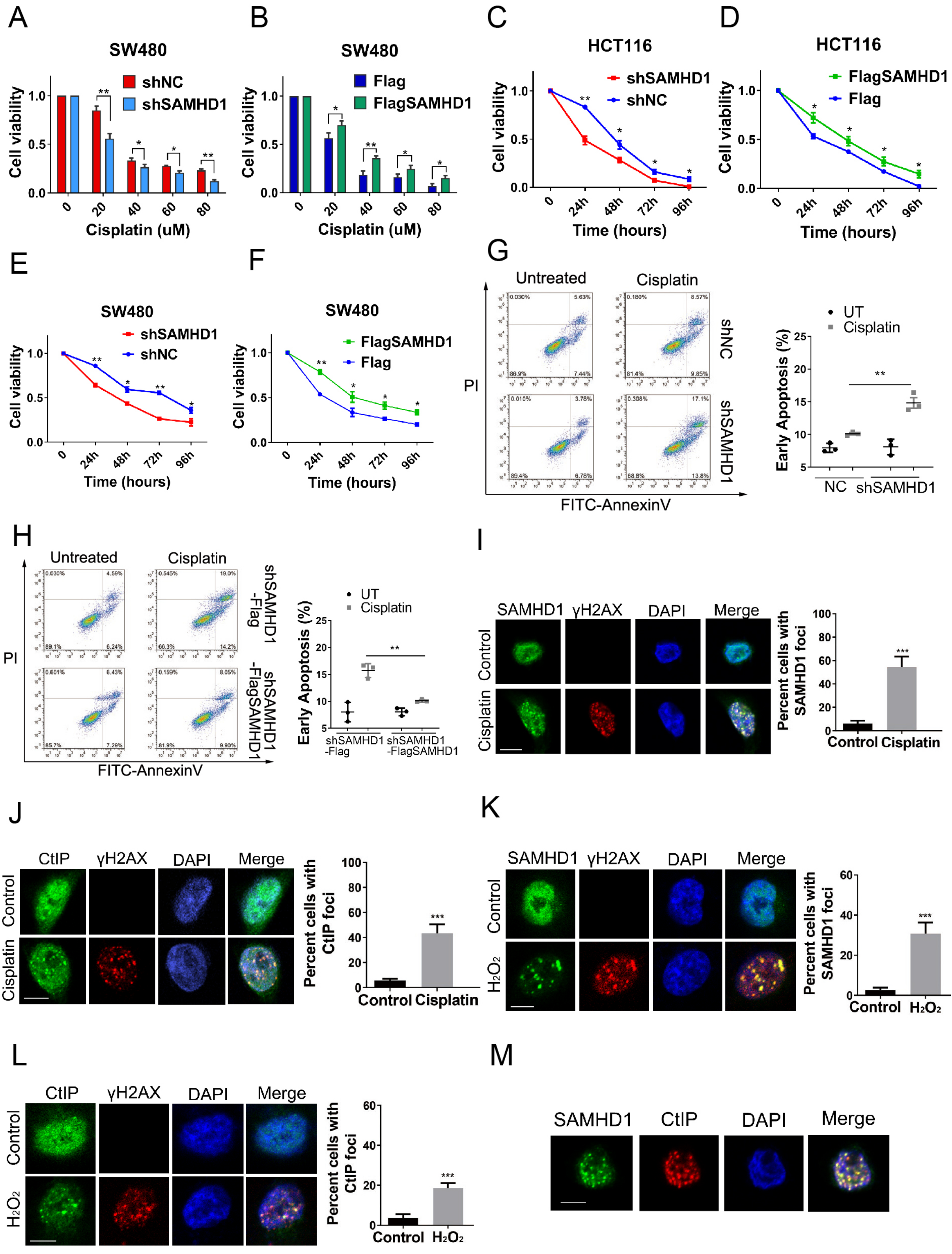

A
SW480
shNC
shSAMHD1
Cell viability
Cisplatin (uM)
B
SW480
Flag
FlagSAMHD1
Cell viability
Cisplatin (uM)
C
HCT116
shSAMHD1
shNC
Cell viability
Time (hours)
D
HCT116
FlagSAMHD1
Flag
Cell viability
Time (hours)
E
SW480
shSAMHD1
shNC
Cell viability
Time (hours)
F
SW480
FlagSAMHD1
Flag
Cell viability
Time (hours)
G
Untreated
Cisplatin
shNC
shSAMHD1
PI
FITC-AnnexinV
UT
Cisplatin
Early Apoptosis (%)
NC
shSAMHD1
H
Untreated
Cisplatin
shSAMHD1 -Flag
shSAMHD1 -FlagSAMHD1
PI
FITC-AnnexinV
UT
Cisplatin
Early Apoptosis (%)
shSAMHD1 -Flag
shSAMHD1 -FlagSAMHD1
I
SAMHD1
γH2AX
DAPI
Merge
Control
Cisplatin
Percent cells with SAMHD1 foci
Control
Cisplatin
J
CtIP
γH2AX
DAPI
Merge
Control
Cisplatin
Percent cells with CtIP foci
Control
Cisplatin
K
SAMHD1
γH2AX
DAPI
Merge
Control
H2O2
Percent cells with SAMHD1 foci
Control
H2O2
L
CtIP
γH2AX
DAPI
Merge
Control
H2O2
Percent cells with CtIP foci
Control
H2O2
M
SAMHD1
CtIP
DAPI
Merge

Figure S4

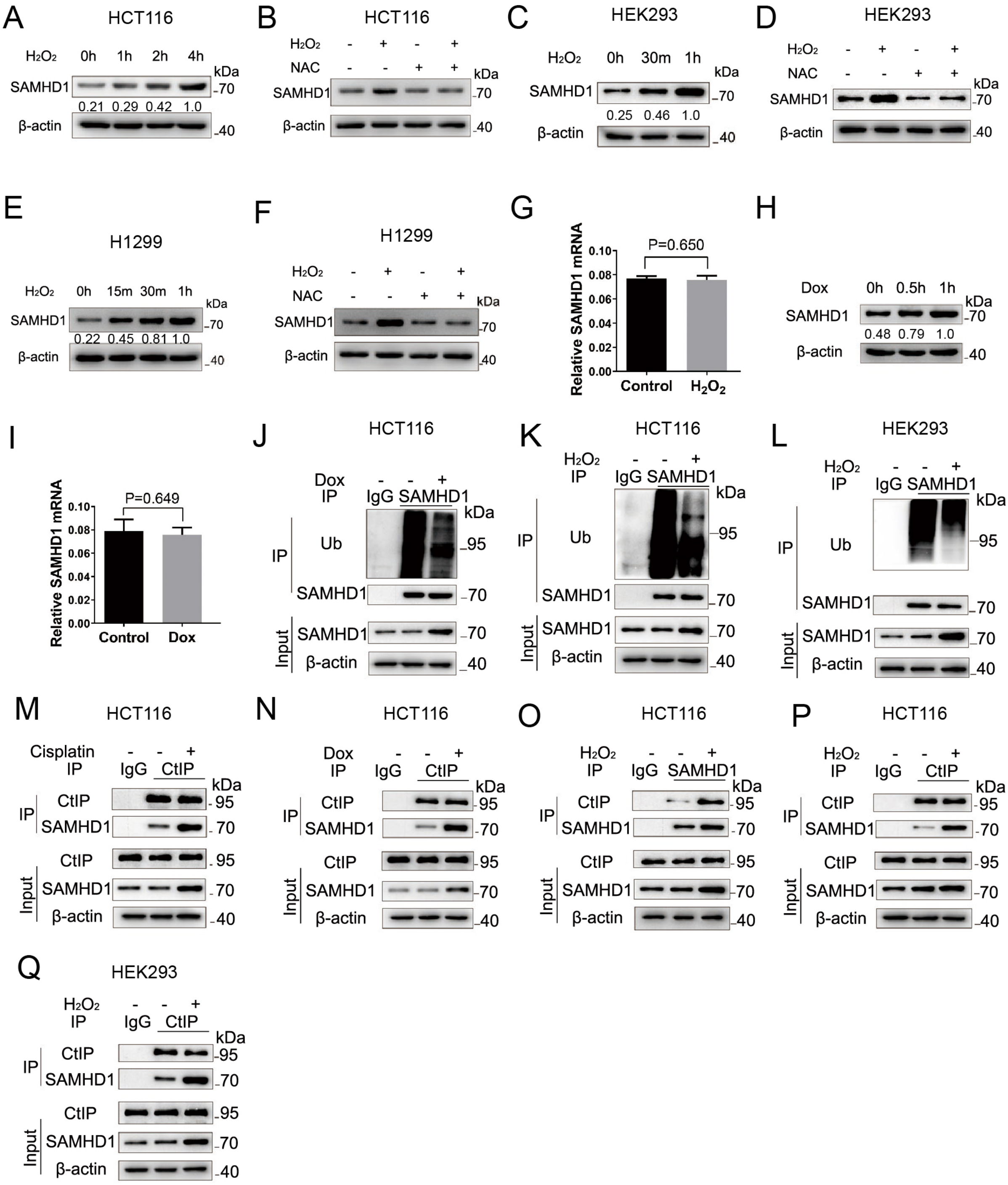
A HCT116
H2O2 0h 1h 2h 4h
SAMHD1 70 kDa
0.21 0.29 0.42 1.0
β-actin 40
B HCT116
H2O2 - + - +
NAC - - + +
SAMHD1 70 kDa
β-actin 40
C HEK293
H2O2 0h 30m 1h
SAMHD1 70 kDa
0.25 0.46 1.0
β-actin 40
D HEK293
H2O2 - + - +
NAC - - + +
SAMHD1 70 kDa
β-actin 40
E H1299
H2O2 0h 15m 30m 1h
SAMHD1 70 kDa
0.22 0.45 0.81 1.0
β-actin 40
F H1299
H2O2 - + - +
NAC - - + +
SAMHD1 70 kDa
β-actin 40
G
P=0.650
Relative SAMHD1 mRNA
0.10 0.08 0.06 0.04 0.02 0.00
Control H2O2
H
Dox 0h 0.5h 1h
SAMHD1 70 kDa
0.48 0.79 1.0
β-actin 40
I
P=0.649
Relative SAMHD1 mRNA
0.10 0.08 0.06 0.04 0.02 0.00
Control Dox
J HCT116
Dox - - +
IP IgG SAMHD1
IP Ub 95 kDa
SAMHD1 70
Input SAMHD1 70
β-actin 40
K HCT116
H2O2 - - +
IP IgG SAMHD1
IP Ub 95 kDa
SAMHD1 70
Input SAMHD1 70
β-actin 40
L HEK293
H2O2 - - +
IP IgG SAMHD1
IP Ub 95 kDa
SAMHD1 70
Input SAMHD1 70
β-actin 40
M HCT116
Cisplatin - - +
IP IgG CtIP
IP CtIP 95 kDa
SAMHD1 70
Input CtIP 95
SAMHD1 70
β-actin 40
N HCT116
Dox - - +
IP IgG CtIP
IP CtIP 95 kDa
SAMHD1 70
Input CtIP 95
SAMHD1 70
β-actin 40
O HCT116
H2O2 - - +
IP IgG SAMHD1
IP CtIP 95 kDa
SAMHD1 70
Input CtIP 95
SAMHD1 70
β-actin 40
P HCT116
H2O2 - - +
IP IgG CtIP
IP CtIP 95 kDa
SAMHD1 70
Input CtIP 95
SAMHD1 70
β-actin 40
Q HEK293
H2O2 - - +
IP IgG CtIP
IP CtIP 95 kDa
SAMHD1 70
Input CtIP 95
SAMHD1 70
β-actin 40

Figure S5

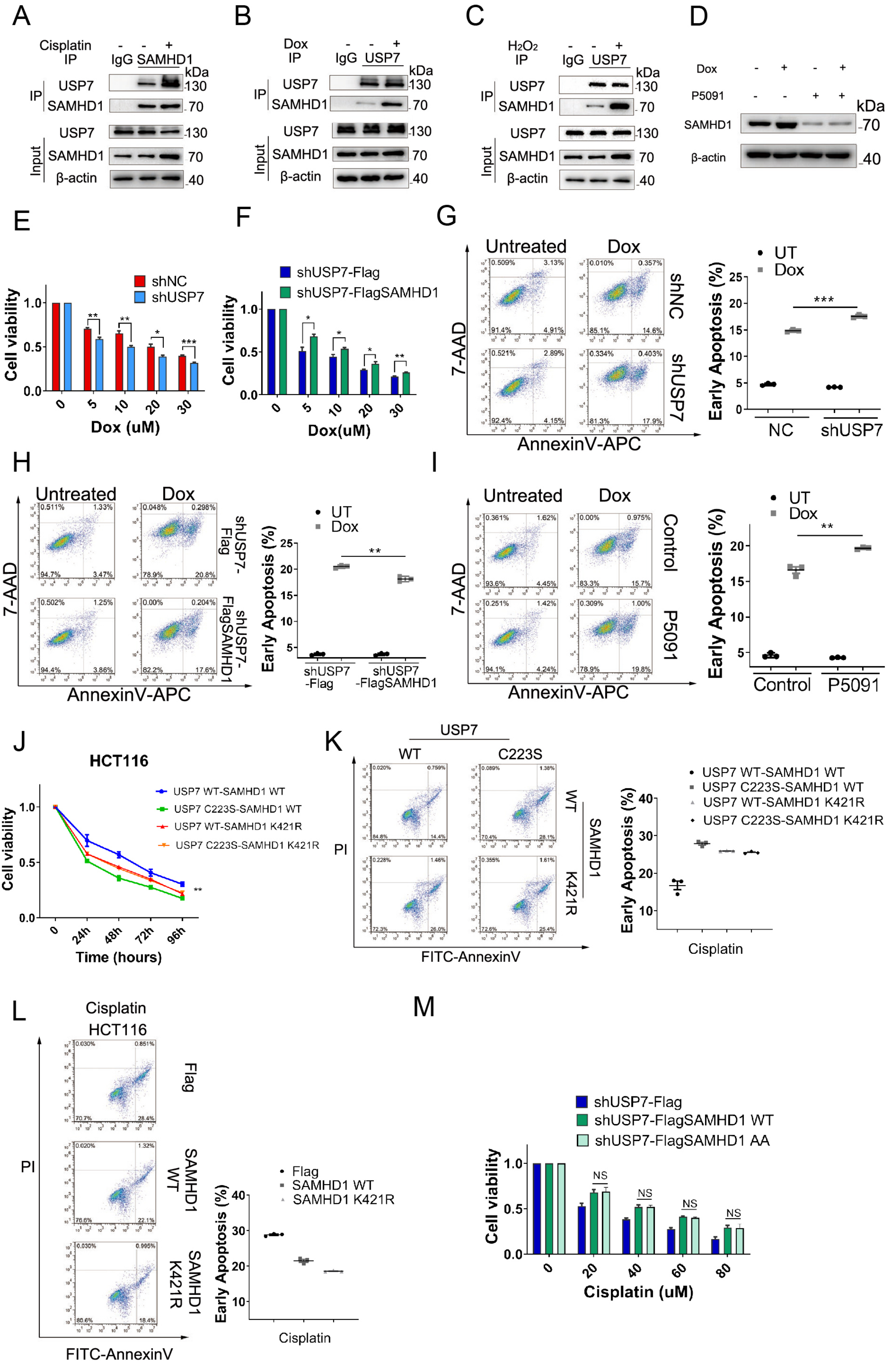

A
Cisplatin
IP
IgG
SAMHD1
kDa
USP7
SAMHD1
IP
Input
β-actin
130
70
40
B
Dox
USP7
C
H_2O_2
D
P5091
E
shNC
shUSP7
Cell viability
Dox (uM)
F
shUSP7-Flag
shUSP7-FlagSAMHD1
Dox(uM)
G
Untreated
7-AAD
AnnexinV-APC
UT
Early Apoptosis (%)
NC
H
I
Control
J
HCT116
USP7 WT-SAMHD1 WT
USP7 C223S-SAMHD1 WT
USP7 WT-SAMHD1 K421R
USP7 C223S-SAMHD1 K421R
Time (hours)
K
WT
C223S
PI
K421R
FITC-AnnexinV
L
Flag
SAMHD1 WT
SAMHD1 K421R
M
shUSP7-FlagSAMHD1 WT
shUSP7-FlagSAMHD1 AA
NS
Cisplatin (uM)

Figure S6

A

Colon adenocarcinoma	SAMHD1 expression Low	Medium	High	n	P
Tumor	4	15	11	30	<0.001
Peritumor	25	4	1	30	

B

Lung adenocarcinoma	SAMHD1 expression Low	Medium	High	n	P
Tumor	8	25	15	48	<0.001
Peritumor	33	12	3	48	

C

Thyroid carcinoma	SAMHD1 expression Low	Medium	High	n	P
Tumor	13	23	22	58	<0.001
Peritumor	49	9	0	58	

D

Colon adenocarcinoma	USP7 expression Low	Medium	High	n	P
Tumor	10	11	9	30	<0.001
Peritumor	28	1	1	30	

E

Lung adenocarcinoma	USP7 expression Low	Medium	High	n	P
Tumor	11	29	8	48	<0.001
Peritumor	34	12	2	48	

F

Thyroid carcinoma	USP7 expression Low	Medium	High	n	P
Tumor	2	25	31	58	<0.001
Peritumor	38	19	1	58	

Figure S7

A

Colon adenocarcinoma	SAMHD1 expression Low	High	n	HR	95%CI	P
Live	78	45	123	2.15	1.07-4.32	0.020
Death	15	19	34			

B

Lung adenocarcinoma	SAMHD1 expression Low	High	n	HR	95%CI	P
Live	22	86	108	3.34	1.65-6.74	0.030
Death	3	62	65			

C

Glioblastoma	SAMHD1 expression Low	High	n	HR	95%CI	P
Live	4	23	27	1.99	1.17-3.38	0.034
Death	9	87	96			

D

Glioma	SAMHD1 expression Low	High	n	HR	95%CI	P
Live	142	39	181	1.56	1.00-2.43	0.026
Death	76	35	111			

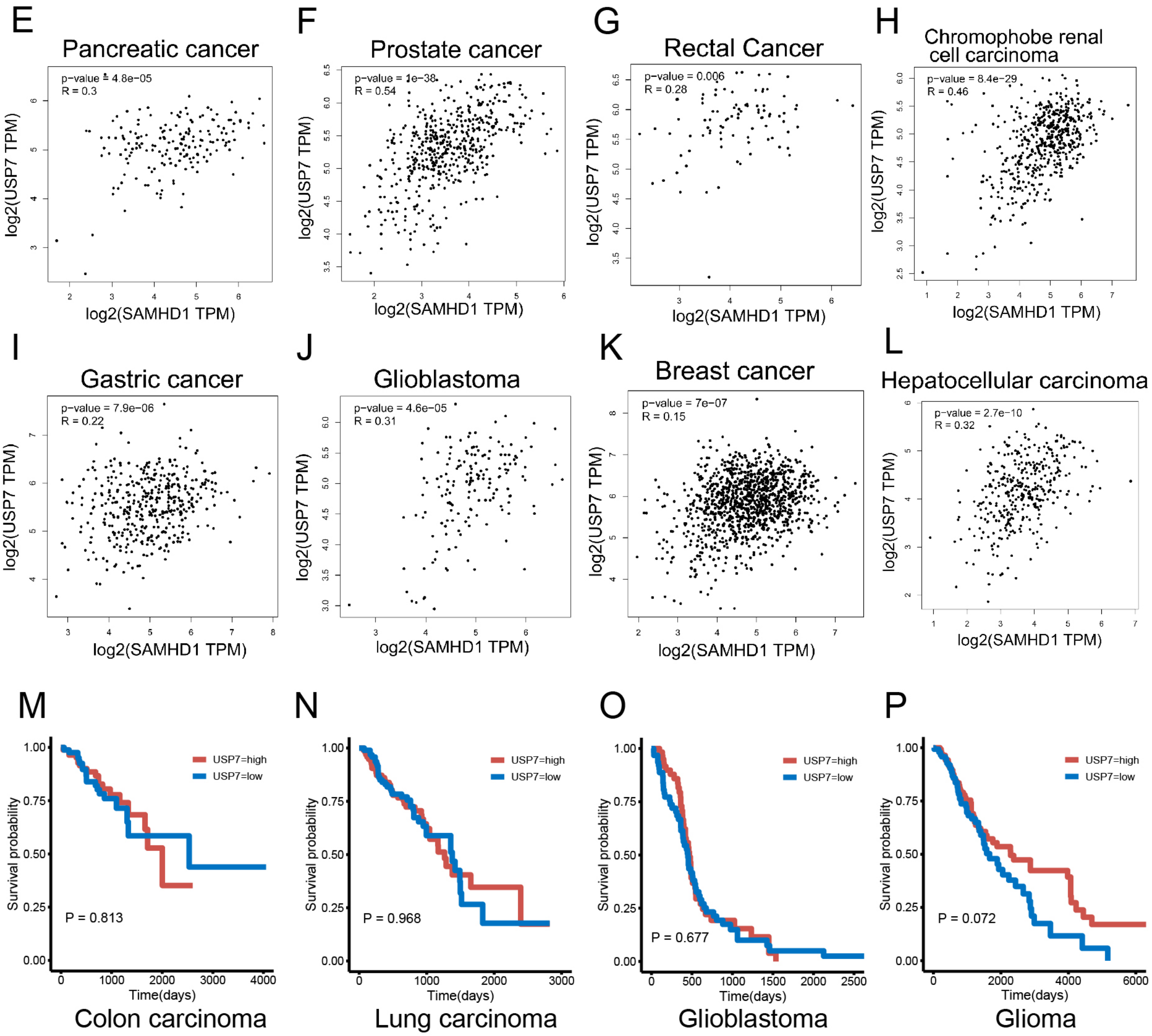

Supplement: Supplementary file 1 — Supplementary Figures [file 41388_2023_2667_MOESM1_ESM.pdf]
