# Supplementary material for: De-ubiquitination of SAMHD1 by USP7 promotes DNA damage repair to overcome oncogenic stress and affect chemotherapy sensitivity
Source: Oncogene. 2023 Apr 20;42(22):1843–56. doi: 10.1038/s41388-023-02667-w (PMC10238270; doi:10.1038/s41388-023-02667-w)
Supplement: Supplementary file 2 — Supplementary Figure Legends [file 41388_2023_2667_MOESM2_ESM.docx]

**Supplementary Figure Legends**

**Figure S1. USP7 binds to SAMHD1 and stabilizes its protein expression.**

**A,** The lysates of Hela cells were immunoprecipitated with the IgG control or anti-SAMHD1 antibody followed by immunoblotting with the USP7 and SAMHD1 antibodies. **B,** The lysates of HEK293 cells were immunoprecipitated with the IgG control or anti-SAMHD1 antibody followed by immunoblotting with the USP7 and SAMHD1 antibodies. **C-D,** The lysates of A549 cells were immunoprecipitated with the IgG control or anti-SAMHD1/anti-USP7 antibody followed by immunoblotting with the USP7 and SAMHD1 antibodies. **E-F,** The lysates of TPC-1 cells were immunoprecipitated with the IgG control or anti-SAMHD1/anti-USP7 antibody followed by immunoblotting with the USP7 and SAMHD1 antibodies. **G-H,** Western blot analysis of SAMHD1 protein level with Flag-USP7 plasmids transfected in A549 or TPC-1 cells. **I,** The lysates of HCT116 cells with Flag or Flag-USP7 transfection for 48h, with or without cisplatin (5μM, 1h) stimulation were subjected to western blot analysis of SAMHD1. **J,** Western blot analysis of SAMHD1 in H1299 cells without or with P5091treatment at different concentrations for 24 h. **K,** Western blot analysis of SAMHD1 in HCT116 cells without or with 20μM of P5091 treatment for different durations. **L-N,** The lysates of HEK293, A549 or TPC-1 cells transfected with or without Flag-USP7 for 48h were immunoprecipitated with the IgG control or anti-SAMHD1 antibody followed by immunoblotting with the ubiquitin antibody. **O-Q,** The lysates of SW480 cells , Hela cells and HEK293 cells with or without P5091 (20μM) pretreatment for 24h were immunoprecipitated with the IgG control or anti-SAMHD1 antibody followed by immunoblotting with the ubiquitin antibody. **R,** The lysates of HCT116 cells were lysed 48 h after transfection with Flag or Flag-USP7 with or without cisplatin (5μM, 1h) stimulation were immunoprecipitated with the SAMHD1 antibody, followed by immunoblotting with the ubiquitin antibody.

**Figure S2. USP7 deubiquitiantes SAMHD1 at K421.**

**A-C,** Mass spectrogram of ubiquitination sites of SAMHD1 K421, K492 and K494. **D,** Sequence alignment of SAMHD1 homologs surrounding K421 in various species. The letter K in red indicates the lysine residues at 421 of SAMHD1. **E,** The lysates of HCT116 cells transfected with Flag-SAMHD1 WT/Flag-SAMHD1 K421R/Flag-SAMHD1 K492R/Flag-SAMHD1 K494R, HA-Ub for 48h were immunoprecipitated with the Flag antibody followed by immunoblotting with the HA antibody. **F,** The HCT116 cells were lysed 48 h after transfection with or without HA-USP7, and Flag SAMHD1 WT or K421R, followed by immunoblotting with the Flag and HA antibody. **G,** The HCT116 cells were lysed 48 h after transfection with or without si-USP7, and Flag SAMHD1 WT or K421R, followed by immunoblotting with the Flag and USP7 antibody. **H and I,** The lysates of HCT116 cells were lysed 48 h after transfection with Flag-SAMHD1 WT/Flag-SAMHD1 K421R, HA-Ub and Myc/Myc-TRIM21/Myc-DCAF1, and then immunoprecipitated with the Flag antibody, followed by immunoblotting with the HA antibody. **J,** The lysates of HCT116 cells were lysed 48 h after transfection with Flag-SAMHD1 WT/Flag-SAMHD1 K622R, HA-Ub and Myc/Myc-USP7, and then immunoprecipitated with the Flag antibody, followed by immunoblotting with the HA antibody.

**Figure S3. SAMHD1 promotes cell survival and reduces apoptosis under genotoxic insults**

**A-B,** SW480 shNC and shSAMHD1 cells or shSAMHD1-Flag and shSAMHD1-Flag-SAMHD1 cells were treated with cisplatin at different concentrations for 24h. Cell viability was assessed by CCK8 assay. **C-F,** HCT116 or SW480 shNC and shSAMHD1 cells or shSAMHD1-Flag and shSAMHD1-Flag-SAMHD1 cells were treated with cisplatin (20μM) for different durations. Cell viability was assessed by CCK8 assay. **G-H,** SW480 shNC and shSAMHD1 cells or shSAMHD1-Flag and shSAMHD1-Flag-SAMHD1 cells were treated with cisplatin (20μM) for 24h followed by PI and FITC-Annexin V staining and FACS analysis. Scatter graph represents percentage of apoptotic cells from three independent experiments. **I,** HCT116 cells with or without cisplatin (5μM) stimulation for 4 h were stained with anti-SAMHD1 and anti-γH2AX antibodies, DAPI, respectively, for immunofluorescence analysis. Scale bar, 10μm. **J,** HCT116 cells with or without 4h of cisplatin (5μM) stimulation were stained with the anti-CtIP and anti-γH2AX antibodies, DAPI, respectively, for immunofluorescence analysis. Scale bar, 10μm. Data are expressed as mean ± SEM. *P < 0.05; **P<0.01; ***P < 0.001. **K and L,** HCT116 cells with and without 1h H_2_O_2_ (500μM) stimulation were stained with the anti-SAMHD1 or anti-CtIP and anti-γH2AX antibodies, DAPI, respectively, for confocal immunofluorescence analysis. Scale bar, 10μm. **M,** HCT116 cells with and without 4h cisplatin (5μM) stimulation were stained with the anti-SAMHD1, anti-CtIP antibodies, and DAPI, for confocal immunofluorescence analysis. Scale bar, 10μm.

**Figure S4. ROS and genotoxic insults increase SAMHD1 expression by deubiquitination for DNA damage repair.**

**A,** Western blot analysis of SAMHD1 in HCT116 cells with and without H_2_O_2_ (25μM) treatment. **B,** Western blot analysis of SAMHD1 in HCT116 cells pretreated with or without NAC (3 mM), followed by 2h of H_2_O_2_ (25μM) stimulation or the vehicle control. **C,** Western blot analysis of SAMHD1 in HEK293 cells with and without H_2_O_2_ (25μM) treatment. **D,** Western blot analysis of SAMHD1 in HEK293 cells pretreated with or without NAC (3 mM), followed by 1h of H_2_O_2_ (25μM) stimulation or the vehicle control. **E,** Western blot analysis of SAMHD1 in H1299 cells without and with H_2_O_2_ (25μM) treatment. **F,** Western blot analysis of SAMHD1 in H1299 cells in the presence and absence of NAC (3 mM) pretreatment, with and without H_2_O_2_ (25μM) stimulation for 1h. **G,** Quantitative PCR for SAMHD1 mRNA in HCT116 cells with or without H_2_O_2_ (25μM) treatment for 2h. **H,** Western blot analysis of SAMHD1 in HCT116 cells with or without doxorubicin (5μM) treatment. **I,** Quantitative PCR for SAMHD1 mRNA in HCT116 cells with or without doxorubicin (5μM) treatment for 1h. **J,** The lysates of HCT116 cells in with or without 1h of doxorubicin (5μM) stimulation were immunoprecipitated with the IgG control or anti-SAMHD1 antibody, followed by immunoblotting with the ubiquitin antibody. **K,** The lysates of HCT116 cells without and with 1h H_2_O_2_ (25μM) stimulation were immunoprecipitated with control IgG or anti-SAMHD1 antibody followed by immunoblotting with ubiquitin antibody. **L,** The lysates of HEK293 cells in with or without 1h of H_2_O_2_ (25μM) stimulation were immunoprecipitated with the IgG control or anti-SAMHD1 antibody, followed by immunoblotting with the ubiquitin antibody. **M and N,** The lysates of HCT116 cells with or without 1h stimulation with cisplatin (5μM) or doxorubicin (5μM) were immunoprecipitated with the IgG control or anti-CtIP antibody, followed by immunoblotting with the SAMHD1 and CtIP antibodies. **O,** The lysates of HCT116 cells with or without 1h stimulation with H_2_O_2_ (25μM) were immunoprecipitated with the IgG control or anti-SAMHD1 antibody, followed by immunoblotting with the SAMHD1 and CtIP antibodies. **P,** The lysates of HCT116 cells with or without 1h stimulation with H_2_O_2_ (25μM) were immunoprecipitated with the IgG control or anti-CtIP antibody, followed by immunoblotting with the SAMHD1 and CtIP antibodies. **Q,** The lysates of HEK293 cells with or without 1h stimulation with H_2_O_2_ (25μM) were immunoprecipitated with the IgG control or anti-CtIP antibody, followed by immunoblotting with the SAMHD1 and CtIP antibodies.

**Figure S5. The USP7-SAMHD1 axis modulates cell survival and apoptosis under genotoxic insults**

**A,** The lysates of HCT116 cells with or without cisplatin (5μM) stimulation for 1h were immunoprecipitated with the anti-SAMHD1 antibody followed by immunoblotting with the SAMHD1 and USP7 antibodies. **B,** The lysates of HCT116 cells with or without doxorubicin (5μM) stimulation for 1h were immunoprecipitated with the anti-USP7 antibody followed by immunoblotting with the SAMHD1 and USP7 antibodies. **C,** The lysates of HCT116 cells with or without H_2_O_2_ (25μM) stimulation for 1h were immunoprecipitated with the anti-USP7 antibody followed by immunoblotting with the SAMHD1 and USP7 antibodies. **D,** Western blot analysis of SAMHD1 in HCT116 cells pretreated with or without P5091 (20μM) followed by stimulation with doxorubicin (5 μM) and the vehicle control for 1h. **E and F,** HCT116 shNC and shUSP7 cells or shUSP7-Flag and shUSP7-Flag-SAMHD1 cells were treated with doxorubicin at different concentrations for 24h. Cell viability was assessed by CCK8 assay. **G and H,** HCT116 shNC and shUSP7 cells or shUSP7-Flag and shUSP7-Flag-SAMHD1 cells were treated with doxorubicin (10μM) for 24h followed by 7-AAD and Annexin V-APC staining and FACS analysis. **I,** HCT116 cells treated with doxorubicin (10μM) for 24h in the presence or absence of P5091 (20 μM) administration followed by with 7-AAD and Annexin V-APC staining and FACS analysis. Scatter graph represents percentage of apoptotic cells from three independent experiments. Data are expressed as mean ± SEM. *P < 0.05; **P < 0.01; ***P < 0.001. **J,** HCT116 cells transfected with USP7 WT/C223S and SAMHD1 WT/K421R were treated with cisplatin (20μM) for different durations. Cell viability was assessed by CCK8 assay. **K,** HCT116 cells transfected with USP7 WT/C223S and SAMHD1 WT/K421R were treated with cisplatin (20μM) for 24h followed by PI and Annexin V-APC staining and FACS analysis. **L,** HCT116 shUSP7 cells transfected with Flag, Flag-SAMHD1 WT or Flag-SAMHD1 K412R cells were treated with cisplatin (20μM) for 24h followed by staining with PI and FITC-Annexin V, and analyzed by FACS. **M,** HCT116 shUSP7-Flag and shUSP7-Flag-SAMHD1 WT and shUSP7-Flag-SAMHD1 H206A/D207A cells were treated with cisplatin at different concentrations for 24h. Cell viability was assessed by CCK8 assay.

**Figure S6. SAMHD1 and USP7 are highly expressed in carcinomas of various organs**

**A-C,** Semiquantitative analyses of SAMHD1 expression in human colonic adenocarcinoma (A, n=30), lung adenocarcinoma (B, n=48) and thyroid carcinoma tissues (C, n=58), and their paired peritumoral tissues. ***P < 0.001 (Mann-Whitney test). **D-F,** Semiquantitative immunohistochemical analysis of USP7 expression in colonic adenocarcinoma (A, n=30), lung adenocarcinoma (B, n=48), thyroid carcinoma (C, n=58) and their paired peritumoral tissues. ***P < 0.001 (Mann-Whitney test).

**Figure S7. SAMHD1 is associated with a worse survival in patients receiving chemotherapy while USP7 positively correlates with SAMHD1 in carcinomas of various origins**

**A-D,** Survival analysis of data from TCGA on the survival outcomes in patients with colonic adenocarcinoma (n=157), lung adenocarcinoma (n=173), glioblasma (n=123) and glioma (n=292) who received chemotherapy. **E-L,** Correlation between USP7 and SAMHD1 expressions in various malignant human neoplasms based on the GEPIA database. **M-P,** Association between USP7 expression and survival outcome in patients with carcinoma of certain organs who received chemotherapy according to The Cancer Genome Atlas (TCGA) data.
